# Generalized structural equations improve sexual-selection analyses

**DOI:** 10.1371/journal.pone.0181305

**Published:** 2017-08-15

**Authors:** Sonia Lombardi, Giacomo Santini, Giovanni Maria Marchetti, Stefano Focardi

**Affiliations:** 1 Department of Biology, University of Florence, Sesto Fiorentino, Florence, Italy; 2 National Research Council-ISC (The Institute for the Complex Systems), Sesto Fiorentino, Florence, Italy; 3 Department of Statistics, Computer Science, Applications, University of Florence, Florence, Italy; Institute of Animal Science, CZECH REPUBLIC

## Abstract

Sexual selection is an intense evolutionary force, which operates through competition for the access to breeding resources. There are many cases where male copulatory success is highly asymmetric, and few males are able to sire most females. Two main hypotheses were proposed to explain this asymmetry: “female choice” and “male dominance”. The literature reports contrasting results. This variability may reflect actual differences among studied populations, but it may also be generated by methodological differences and statistical shortcomings in data analysis. A review of the statistical methods used so far in lek studies, shows a prevalence of Linear Models (LM) and Generalized Linear Models (GLM) which may be affected by problems in inferring cause-effect relationships; multi-collinearity among explanatory variables and erroneous handling of non-normal and non-continuous distributions of the response variable. In lek breeding, selective pressure is maximal, because large numbers of males and females congregate in small arenas. We used a dataset on lekking fallow deer (*Dama dama*), to contrast the methods and procedures employed so far, and we propose a novel approach based on Generalized Structural Equations Models (GSEMs). GSEMs combine the power and flexibility of both SEM and GLM in a unified modeling framework. We showed that LMs fail to identify several important predictors of male copulatory success and yields very imprecise parameter estimates. Minor variations in data transformation yield wide changes in results and the method appears unreliable. GLMs improved the analysis, but GSEMs provided better results, because the use of latent variables decreases the impact of measurement errors. Using GSEMs, we were able to test contrasting hypotheses and calculate both direct and indirect effects, and we reached a high precision of the estimates, which implies a high predictive ability. In synthesis, we recommend the use of GSEMs in studies on lekking behaviour, and we provide guidelines to implement these models.

## Introduction

Sexual selection is a fundamental evolutionary force that operates either through (i) direct competition between males or (ii) female mate choice which leads to the evolution of forms of exaggerated and useless ornaments in males (e.g. the peacock’s tail). The ornaments are supposed to display male genetic quality or the absence of sexually transmissible diseases [1]. Albeit a long record of studies since Darwin’s time have addressed this problem, many questions about sexual selection remain open, and this continues to be a major research theme. For the present contribution, the main question is how to investigate the factors affecting male copulatory success in lek mating. In lekking species, the two sexes interact mainly during the rut [2, 3] when males defend small display territories inside an arena or lek. For males, lekking is a high cost—high benefits strategy, in which the risk of injuries and even death is high, but a few dominant males may monopolize most of the copulations [4, 5]. On the other hand, females are supposed to benefit from visiting a lek, since they can choose among several potential partners [6, 7].

Lekking has been described in many different taxa (reviewed by Hoglund & Alatalo [3]) such as insects, fishes, amphibians, reptiles, birds [3, 8, 9, 10] and mammals [2, 7, 11]. In leks, male mating success is highly skewed [12]. However this features is not unique to leks and it is found in other reproductive systems as well [13]

As a specific example, in fallow deer (*Dama dama*) [11, 14], the breeding system is highly variable and lekking is not the only strategy [5, 15]. Independtly from the breeding system in this species the skew of male copulatory success appear always very high. Two main hypotheses have been proposed to explain the observed asymmetry in copulatory success: female choice, FCH, and male dominance, MDH, [16, 17, 18]. FCH [19] assumes that the females select mates on the basis of the phenotypic traits of males, while according to MDH the copulatory success is determined by lek attendance and a high dominance rank [20] In fallow deer, several studies pointed out that female choice is the most likely determinant of copulatory skew [15, 21, 22, 23]. However, Clutton-Brock et al. 11] argue that copulatory success may not be solely related to female preferences for specific male traits, but it may also arise from different reasons, such as the need to minimize the risk of predation or harassment. Other authors, on the contrary, suggested that copulatory success strictly depends on male dominance rank [5, 24, 25, 26, 27, 28].

A number of different statistical techniques have been used to investigate the copulatory success in lekking species (e.g. [12]). Most papers have applied standard linear models (e.g., [12, 29, 30]), mixed models to account for repeated observations (e.g. [31, 32]), or Generalized Linear Models to manage non-normal distributions. Finally, a few papers have used different approaches, such as logistic regression [33], path analysis [34], and partial correlations [35]. A detailed list of the methods used in the literature is reported in S2 Table. A critical reading of this literature puts into light several methodological shortcomings: i) multicollinearity among explanatory variables [35], (ii) erroneous handling of non-normal and non-continuous distributions of the response variable, and (iii) problems in inferring cause-effect relationships, so that no firm decision on the prevalence of female choice or male dominance could be established [34].

Multicollinearity, which occurs when two or more predictors in a multiple regression model are highly correlated, leads to variance inflation and increase type-I errors, thus making some of the coefficients appear significant when they are not [36].

Another important source of bias depends on erroneous handling of non-normal and non-continuous distributions of the response variable. Copulatory success is a classic example of such a variable; in leks, only a few males have access to mating, and this process leads to a zero-inflated distribution of copulations. In many cases, this problem is dealt with using square root or logarithm transformations [12, 33, 35, 37], but despite this procedure being recommended in general biometry textbooks (e.g., [38]), its validity is restricted to cases when deviations from normality are only to limited extent. Moreover, discrete response variables containing many zeros cannot be transformed into normal distributions, and inference is doomed to be severely biased [39, 40].

There are concerns related to the link between correlations and causation, which are tricky to deal with. Explanatory variables and copulatory success may, in fact, appear unrelated when they are related, or on the contrary, they may be correlated even when no causal link is present. A spurious or missing correlation may arise for several reasons which include (i) a common causation that induces a false relationship or cancels out an existing association, (ii) a reciprocal association loop, (iii) a conditional relationship between explanatory and response variables following the value of a third control variable, or (iv) a non-linear association between dependent and independent variables [41, 42, 43, 44]. When a correlation between two variables is detected, cause-effect relationships cannot be easily deduced without further assumptions [45,41]. The best way to test causal relationships is to use a proper experimental design where the hypothetical cause is directly manipulated [45]. However, manipulative experiments are difficult to achieve, and researchers have to rely mainly on observational studies [12, 3, 46].

The problem of inferring cause-effect relationships among variables can be addressed by path analysis or Structural Equation Models (SEM) [47]. In field studies often the variables of interest cannot be directly recorded by the observers. For instance, we cannot measure the “sex appeal” of males [48]. However, we can measure some traits we expect to be correlated to “sex appeal” and so obtain an indirect evaluation of the variable of interest. This is the same done in principal component analysis: a reduced number of meaningful factors are estimated from the correlations among a large number of descriptors. In SEM terminology, we refer to the unobservable factors as latent and to the observed descriptors as manifest (a detailed discussion is presented in S3 Text and in S3 Fig). A SEM is a combination of a measurement model that defines latent variables using one or more manifest variables and a structural model that imputes causal relationships between latent variables [41]. The development of a measurement model is also important to control for the errors introduced during observations, i.e., it represents a state space model for the unobserved variables of interest. In this way, a latent variable is not directly observed, but its existence is inferred by the way it influences manifest variables that can be directly observed [41].

One known limitation of standard SEM is to assume that all variables are normally distributed [49]. The introduction of Generalized Structural Equations Models (GSEM), may overcome this limitation. In GSEM, it is possible to have a model with both continuous and discrete variables grouped together in the same latent construct. As such, GSEM combines the power and flexibility of both SEM and GLM in a unified modeling framework. The advantages of GSEM are: (i) to evaluate potential causal relationships with the “structural model”; (ii) to consider both direct and indirect effects of multiple interacting factors, simultaneously [41, 47, 50, 51]; (iii) the possibility of using appropriate probability density functions other than the normal one for manifest indicators and latent constructs.

In this paper, we contrast the main statistical methods used in literature to GSEM using data from a specific study case about fallow deer lekking behaviour. First, we reviewed the available literature on lekking behaviour to obtain an overview of the statistical methods used. Secondly, we fitted the main types of models used. Third, within a SEM framework, we formulated two models, one describing the FCH and the other the MDH hypotheses, and fitted them using both SEM and GSEM, for comparison. Finally, we compared the predictive performances of the different methods using information theoretic indexes (AIC and BIC), residual analysis, and precision of regression coefficients.

## Materials and methods

### Study area and data collection

Field observations were carried out during 1991 and 1992 ruts (September-October) in the Preserve of Castelporziano near Rome (Italy) (coordinate), an area covering 42 km^2^. The habitat is characterized by an old-growth natural oak wood, with both evergreen *(Quercus ilex* and *Q*. *suber)* and deciduous (mainly *Q*. *cerris* and *Q*. *frainetto*) tree species. A detailed description of the vegetation of the study area can be found in Bianco et al. [52]. Information on ungulate populations are given in Focardi et al. [53] and Imperio et al. [54]. The dataset was used to estimate two different dominance indexes: (a) *Dom* [55]; (b) David’s score, *Ds* [56]. To obtain index values comparable across years, *Dom* and *Ds* were relativized to the number of fights observed in each year. The number of observed copulations achieved by a buck in one rut was used as a measure of copulatory success (*CopS*).

Two measures of lek attendance were computed: *LA*_*1*_, is the number of total days in which an animal was seen at the lek and *LA*_*2*_ is the number of days the animal was able to hold a territory. Finally, we estimated the average number of females observed in one buck’s territory (harem size—*HS*) and courtship success (*CourtS*) as the number of courtships terminated with a copulation divided by the total number of attempts (number of copulations /number of courtship events, for every male).

Two variables were used: a) the total number of spellers (*TotS*) and b) a measure of fluctuating asymmetry for small spellers [57, 58] ASS_T_.

Further details on study area, data collection, data validation and measures computations are provided in S1 Text, S1 Table, S1 and S2 Figs in Supporting information.

### Ethic statement

This work does not imply animal handling or capture. The “Segretariato alla Presidenza della Repubblica” was the authority responsible for the permission to work in the Preserve of Castelporziano, Rome, (Italy). The fieldwork was based on a research and management agreement between the I.S.P.R.A -The Italian National Institute for Environmental Protection and Research (ex I.N.F.S. National Institute for Wildlife) (former institution of SF 1988–2011), the Director of the Preserve of Castelporziano, Dr. A. Demichelis, the Preserve research responsible, Dr. A. Tinelli, in collaboration with the Presidential Estate rangers, and the Corpo Forestale dello Stato (C.F.S.) under the combined prescriptions of the Italian law which regulates studies on wild species and does not require that the I.S.P.R.A. obtain permits from any other authorities. The field study did not involve endangered or protected species and this implied that it was not required any approval from Institutional Animal Care and Use Committee. The study was not carried out on private land.

### Statistical analysis

We compared several modelling approaches described in the literature. We were aware that some of these approaches are inherently flawed, but we decided to use them due to their widespread use in the pertinent literature on leks (cfr. S2 Text and S2 Table). All the tested models have *CopS* as the response variable. Note that *CopS* is discrete by definition (because it is a count) and hence cannot be assumed to be normally distributed.

### Linear Models and Generalized Linear Models

The copulatory success of the *i*-th buck (*CopS*) is modelled as:

$$CopS_{i}=\beta_{0}+\beta_{1}x_{1,i}+\beta_{2}x_{2,i}+\ldots +\beta_{p}x_{p,i}+\varepsilon_{CopS}$$ (1)

where the *x*_*p*,*i*_ are predictor variables, the *β*s regression coefficients and *ε*_*CopS*_ is the error term.

Following the approaches described in the literature, we first used ordinary least squares regression where the response variable *CopS*. was untransformed, log-transformed, or square-root transformed. Secondly, we used GLMs for count data. The following models were considered:

LM_1_, multiple regression model without *CopS* transformation;

LM_2_ where the dependent variable is log(*CopS +1*);

LM_3_ where the dependent variable is log(*CopS +0*.*5*);

LM_4_ where the dependent variable is log(*CopS +0*.*1*);

LM_5_ where the dependent variable is *CopS* ^*0*.*5*^;

GLM_1_, Generalized Linear Model where *CopS* follows a Poisson distribution;

GLM_2_, assuming that *CopS* follows a Negative Binomial distribution;

GLM_3_, assuming that *CopS* follows a Zero Inflated Poisson distribution (ZIP);

GLM_4_, assuming that *CopS* follows a Zero Inflated Negative Binomial distribution (ZINB);

GLM_5_, assuming that *CopS* follows a Hurdle at Zero Distribution (Hurdle). In the Hurdle models a Bernoulli probability governs the binary outcome of whether a count variable has a zero or positive realization. When the realization is positive the conditional distribution is modelled by a truncated at zero count data model.

For each type of model we considered both the full model, which includes all significant (P<0.05) and non-significant coefficients and the Minimal Adequate Models (MAM) which include only significant values [59]. MAMs, hereafter denoted by the suffix *r* (e.g. GLM_4,r_) were obtained using a *p*-value selection procedure [60].

Akaike information criterion (AIC) and Bayesian information criterion (BIC) were also computed to assess model performances.

Statistical analysis was carried out in R [61], using the packages *fitdistrplus*, *gamlss*, *pscl*, *vcd*.

#### Generalized Structural Equation Models

A. A SEM requires the *a-priori* definition of links among model variables in the form of a regression equations system. The goal of this class of models is minimize the difference between estimates and expectations variance-covariance matrix of data.

Latent variables are unobserved factors denoted,*η*_1_,*η*_2_,….,*η*_n_ that represent an hypothetical construct that can be inferred by the way it influences manifest or observed variables (continuous, *Y*_i_ = *y*_*1*_, *y*_*2*_,..,*y*_*n*_) [41, 51].

**Fig 1:**
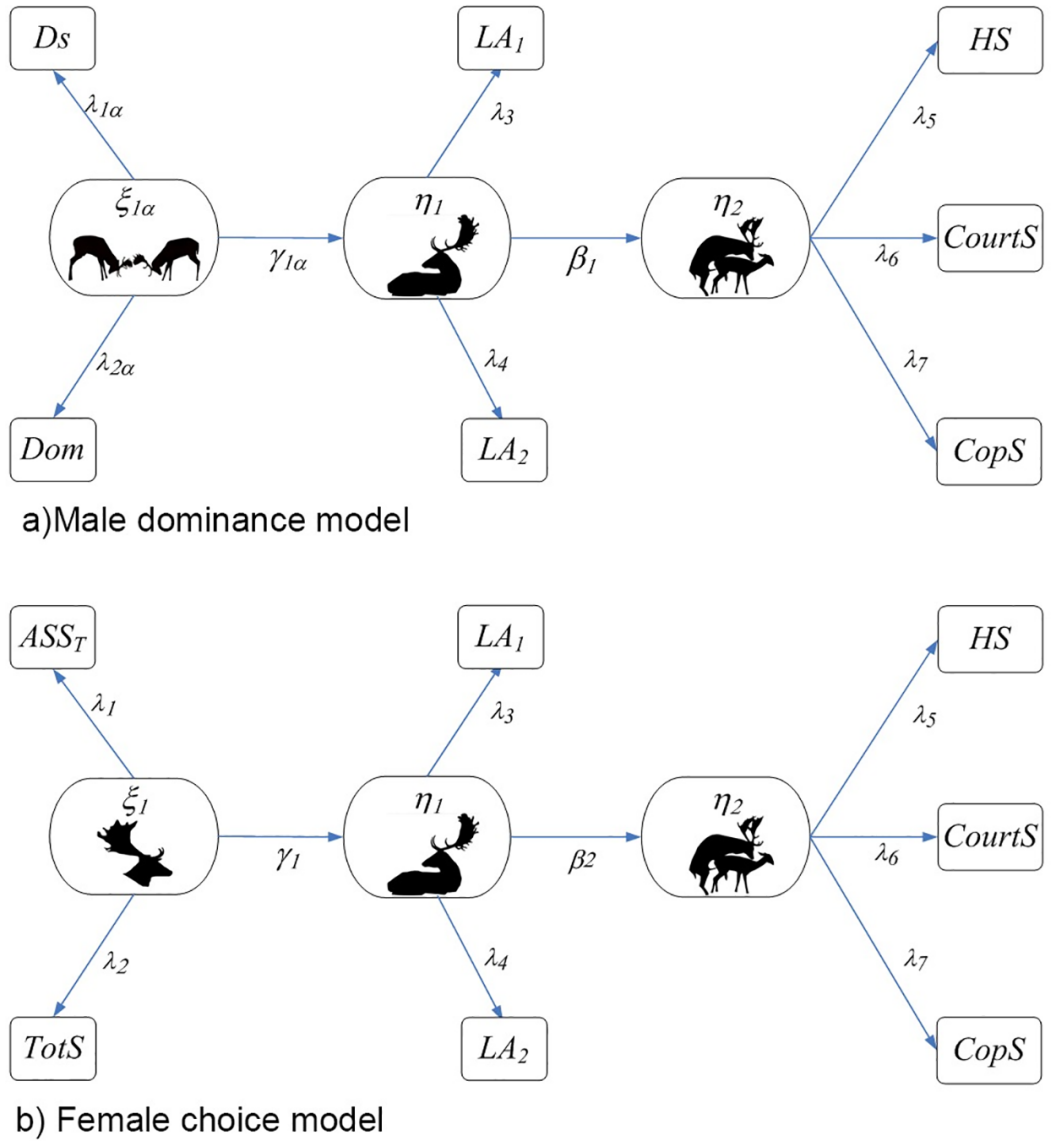
Path diagrams for a) the “dominance male” model (MDH) and b) “female choice” model (FCH). Variable names are: *ASS*_*T*_ = the fluctuating asymmetry of small antler’s spellers; *TotS* = total number of small and large antler’s spellers; *Dom* = Dominance Index (Clutton-Brock Index [55]) divided by the total number of bucks of each year; *Ds* = the David’s score (Gammel et al.) [56] divided for the total number of bucks of each year; *LA*_*1*_ = number of days in which the animal was present in the lek. *LA*_*2*_ = total number of days of presence/territory in different locations of the same lek. *HS* = average number of females in a male’s territory; *CourtS* = the fraction of courtship events terminated with a copulation (number of copulations / number of courtship events, for every male); *CopS* = total copulatory success of the i-th buck in one rut. The number of observations is the same for all models (N = 118). Symbols and variables are described in the text and in S1 Table.

A SEM model is composed by two sub-models: a measurement model that describes the relationships between latent variables and their manifest variables and a structural or causal model that constitutes a directional chain system that describes the hypothetical causal relationship between the constructs of theoretical interest (latent variables) using *path diagrams* (Fig 1a and 1b).

Structural coefficients or regression coefficient (*γ*, *β*, *λ*) represent the effects of each independent variable on the dependent variable (Fig 1a and 1b).

A manifest variable, in a SEM with latent variables, plays a role of endogenous variable if it is predicted by another variable in the model and is therefore a response variable; it is assumed to be generated as a linear function of its latent dimension and the residual error term represents the imprecision in the measurement process. An exogenous variable whose variation is not explained in a model (i.e. fluctuating asimmetry of small spellers *ASS*_*T*_ or *Dom*). A description of SEM modelling is reported in S3 Text, S3 Table and S3 Fig.

GSEMs represent a generalization of SEMs by allowing the use of discrete variables and non-Gaussian distributions. They combine observed (or manifest) and latent variables representing unmeasured constructs. A GSEM [62] reads:

$$\begin{array}{l} \eta =f_{\eta }\left(\eta ,\xi ,\zeta \right)\\ x=f_{x}\left(\eta ,\delta \right)\\ y=f_{y}\left(\eta ,\varepsilon \right) \end{array}$$ (2)

where *x* and *y* are vectors of manifest variables and *η*, *ξ*, *ζ* represent the latent variables, while *δ*, and *ε* denote the error terms. The functions (*f*_*η*,_ *f*_*y*,_ *f*_*x*_) provide a general way to represent the connections between the variables within the parentheses to those on the left hand side of each equation. We developed and compared two different causal models, one assuming that copulatory success is determined by MDH and the other one based on FCH.

We verified that the number of parameters is identifiable according to rules 1 and 3 of Shipley [41]. We used a robust maximum likelihood estimator and a sandwich estimator [63]. We fitted GSEMs with both Mplus [64] and STATA [65]. We used both softwares to check that the results are identical. Further STATA provided case-specific residuals which are not outputted by Mplus. On the other hand, Mplus returns the standardized path coefficients and total, direct, and indirect effects which STATA does not compute. The STATA and Mplus codes used to generate SEM and GSEM models are presented in S4 Text.

#### Models’ comparison

Unfortunately, there is no a simple method for comparing these different sets of models. GLMs and LMs can be compared by AIC or BIC, but only if the dependent variable is not transformed [66]. To overcome this problem and make all LMs and GLMs comparable, we calculated the maximum likelihood estimates from the log-transformed or root square—transformed model applying the formula reported in Weiss [67] (see S5 Text for details).

The comparison of SEM or GSEM with AIC is questionable due to the presence of latent variables which increase AIC values making these models not comparable to GLMs [68]. On the other hand, the use of absolute fitting indexes is vulnerable to criticisms [69, 70]. We compared models by two different approaches. First, we measured the precision of each estimated regression coefficient $\hat{\beta }$ by computing its coefficient of variation ($CV=\frac{SE(\hat{\beta \;)}}{\left|\hat{\beta \;}\right|}=\;\frac{1}{\left|\hat{\beta \;}\right|/SE(\hat{\beta )}}=\frac{1}{\left|T\right|}=\sqrt{\chi_{1}^{2}}$, where T is the statistic test and $\chi_{1}^{2}$ is the chi-square test with one degree of freedom). For a more general evaluation of the model’s precision, we computed the median CV for the parameters estimated by each model [71]. Second, we performed an analysis of case-specific residuals. In principle, if a model correctly fits the data, the residuals are expected to have zero mean, normal distribution, without any pattern or structure. We visually checked residual distributions and computed their mean, variance, and kurtosis. The best distribution is the one with the smallest variance of residuals, symmetrical and centered around zero.

#### Definition of working hypotheses

In this paper, we contrast two working non-nested hypotheses, “male dominance” (MDH) and “female choice” (FCH). The structure of the models corresponding to the Male Dominance Hypothesis (MDH) and the Female Dominance Hypothesis (FDH) is shown in Fig 1. We have assumed, according to literature, the existence of four latent variables: *ξ*_*1*_ represents the effect of antler shape and is described by *ASS*_*T*_ and *TotS*, *ξ*_*1a*_ represents male dominance and is described by *Dom* and *Ds*, *η*_*1*_ represents lek attendance (*LA*_*1*_ and *LA*_*2*_). Finally, *η*_*2*_ represents courtship and is measured by *HS*, *CourtS*, and *CopS*. The use of latent variables allowed us to reduce the unavoidable errors in the measurement of manifest variables. For MDH we assume that *ξ*_*1a*_ influences *η*_*1*_, or in other words the fighting ability of bucks determines their lek attendance and territory holding. Being able to defend a territory allowed a buck to keep a harem and finally to sire females. For the FCH we assume that male phenotypic quality, *ξ*_*1*_, which represents its health and physical fitness, allows the buck to stay in the lek for a long time and to be selected by wandering females.

Note that SEM allows us to study the effects of remote and proximate causes of male copulatory success in the same statistical framework. Further, the use of latent variables reduces the unavoidable errors in the measurement of manifest variables. Once the measurement model is defined, we can establish appropriate causal relationships among latent variables.

The MDH is implemented by the following system of regression equations (Fig 1a):

$$\begin{array}{l} Ds=\lambda_{1a}\xi_{1a}+\delta_{Ds}\\ Dom=\lambda_{2a}\xi_{1a}+\delta_{Dom}\\ LA_{1}=\lambda_{3}\eta_{1}+\epsilon_{LA_{1}}\\ LA_{2}=\lambda_{4}\eta_{1}+\varepsilon_{LA_{2}}\\ HS=\lambda_{5}\eta_{2}+\varepsilon_{HS}\\ CourtS=\lambda_{6}\eta_{2}+\varepsilon_{CourtS}\\ CopS\sim Poisson\left(\mu \right),\text{log}\left(\mu \left(CopS\right)\right)=\lambda_{7}\eta_{2}. \end{array}$$ (3)

The model for FCH is represented in Fig 1b and reads:

$$\begin{array}{l} ASS_{T}=\lambda_{1}\xi_{1}+\delta_{ASS_{T}}\\ TotS=\lambda_{2}\xi_{1}+\delta_{TotS}\\ LA_{1}=\lambda_{3}\eta_{1}+\epsilon_{LA_{1}}\\ LA_{2}=\lambda_{4}\eta_{1}+\varepsilon_{LA_{2}}\\ HS=\lambda_{5}\eta_{2}+\varepsilon_{HS}\\ CourtS=\lambda_{6}\eta_{2}+\varepsilon_{CourtS}\\ CopS\sim Poisson\left(\mu \right),\text{ log}\left(\mu \left(CopS\right)\right)=\lambda_{7}\eta_{2}. \end{array}$$ (4)

FCH and MDH used 21 and 19 free parameters, respectively, which are identifiable, according to Shipley [41].

## Results

**Fig 2:**
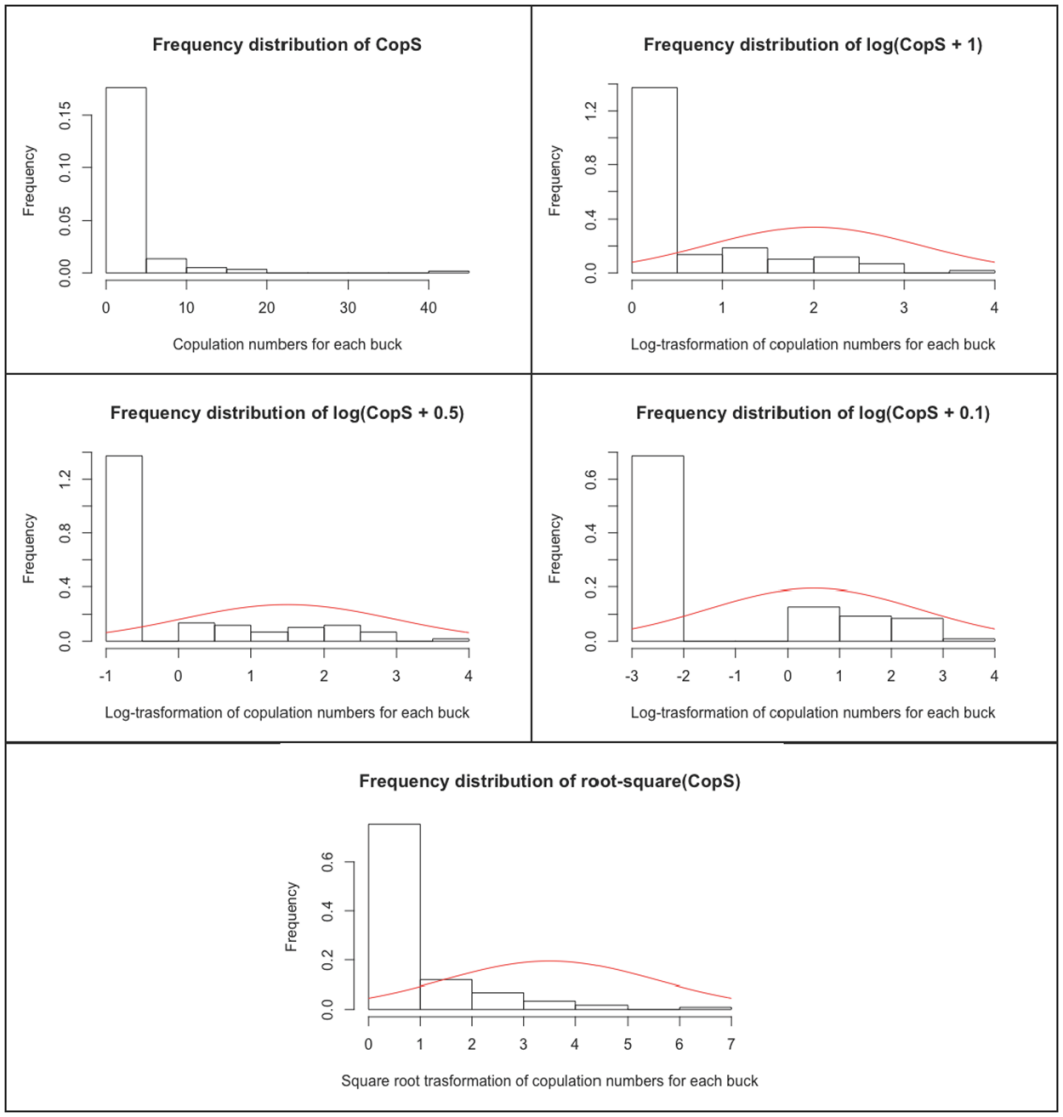
Frequency distribution of number of copulations achieved by each buck (*CopS*) before (upper left panel) and after transformation. The continuous red line shows the theoretical normal curve for reference.

The distribution of *CopS* is showed in Fig 2. Most of the bucks (68.6%) had no copulations. The number of copulations per individual ranged from 0 to 43, and the distribution has high kurtosis (32.33) and skewness (4.99). The distribution of *CopS* is best fitted by a negative binomial distribution (*χ*^2^ = 0.28, *P* = 0.595), which is much better supported than alternative models (ZINB, ΔAIC = 33.39; ZIP, ΔAIC = 152.47; Poisson, ΔAIC = 535.02). Data transformation changes the discrete *CopS* distribution into a continuous one, which remains, however, non-normally distributed (Shapiro-Wilk Test: log(*CopS*+1), *W* = 0.648, P<0.001; log(*CopS*+0.5), *W* = 0.659, P<0.001; log(*CopS*+0.1), *W* = 0.662, P<0.001; *CopS*^0.5^, *W* = 0.635, P<0.001).

### Linear and Generalized Linear Models

The AIC and BIC values associated with LMs with untransformed response variables and GLMs are reported in Table 1. LM_1_ and LM_1r_ have considerably higher AIC and BIC than GLMs. Among the different GLMs, GLM_*2*,*r*_ exhibits the lowest AIC and BIC values, while the corresponding full model, GLM_*5*,*r*_ has higher AIC and BIC values. GLM_*4*,*r*_ presents the same AIC values as GLM_*2*,*r*_, but a higher BIC values. As expected, MAMs show lower fit indexes than corresponding full models, except in Hurdle model. The different models identify different sets of significant variables, and the unstandardized coefficients for all models are given in S4 Table. In synthesis, among the eight variables considered, only *HS* and *CourtS* (except in GLM_*5*,*r*_) are always detected as significant, whereas *TotS*, *Ds*, and *LA*_*1*_ were only put into light by some of the GLMs. Note, however, that their estimates are nonsensical since they are always negative, whereas positive values are expected. This is an example of Simpson’s paradox, which Pearl (e.g. [47]) has discussed as a common problem with non-SEM studies.

**Table 1:** AIC and BIC values associated with linear (untransformed) and GLM models.

Model	Type	K	AIC	BIC
*LM*_*1*_	*Normal*	9	662.6	687.5
*LM*_*1*,*r*_	*Normal*	3	657.7	670.9
*GLM*_*1*_	*Poisson*	9	283.2	308.2
*GLM*_*1*,*r*_	*Poisson*	6	280.2	296.8
*GLM*_*2*_	*Neg Binom*	10	224.7	252.4
*GLM*_*2*,*r*_	*Neg Binom*	4	222.7	233.8
*GLM*_*3*_	*ZIP*	10	285.2	313.0
*GLM*_*3*,*r*_	*ZIP*	7	282.2	309.9
*GLM*_*4*_	*ZINB*	11	226.7	257.2
*GLM*_*4*,*r*_	*ZINB*	5	224.7	255.2
*GLM*_*5*_	*Hurdle*	10	385.3	412.9
*GLM*_*5*,*r*_	*Hurdle*	6	400.8	414.7

K = number of parameters in the model. Type indicates the distribution used. The suffix *r* indicates reduced models

The models with linear transformed response variables (Table 2) have erratic AIC and BIC values varying from a minimum for LM_*4*,r_ (AIC = -67.6 and BIC = -59.3) to a maximum associated to LM_*5*._ (AIC = 347.7 and BIC = 372.6). AIC and BIC values vary in an unpredictable way depending on the value of the constant added to the transformed variable (or in the calculation of maximum likelihood in the case of the square root transformation). Due to the complete unreliability of data transformations, this approach will not be considered further in this paper.

**Table 2:** AIC and BIC values associated with linear models with transformed response variables.

Model	Transformation	K	AIC	BIC
*LM*_*2*_	*log(x+1)*	9	250.2	275.1
*LM*_*2*,*r*_	*log(x+1)*	3	249.4	257.7
*LM*_*3*_	*log(x+0*.*5)*	9	157.5	182.4
*LM*_*3*,*r*_	*log(x+0*.*5)*	3	156.7	165.0
*LM*_*4*_	*log(x+0*.*1)*	9	-66.3	-41.3
*LM*_*4*,*r*_	*log(x+0*.*1)*	3	-67.6	-59.3
*LM*_*5*_	*x* ^*0*.*5*^	9	347.7	372.6
*LM*_*5*,*r*_	*x* ^*0*.*5*^	3	344.7	353.0

K = number of parameters in the model. Transformation indicates the type of transformation applied to the dependent variable. The suffix *r* indicates reduced models.

### Structural Equation Models

The variance-covariance/correlation matrix used in SEM and GSEM is reported in S5 Table.

To select the appropriate distribution of *CopS* for GSEM, we first selected the discrete distributions available both in Mplus and STATA. It resulted that only two of these distributions, Poisson and Negative binomial, were supported. According to the results of Table 1, we first tested the negative binomial distribution, but the model did not converge in either software. Thus we were forced to use the Poisson distribution.

If we implement the SEM for MDH with Mplus, convergence is not achieved, because the residual covariance matrix is not positive definite [72] and the residual variances associated with LA_1_ have negative values. Note that the AIC values yielded by Mplus are biased. Indeed, in GSEM the convergence of the MDH model is only achieved by fixing the path-coefficients for *Dom*, *LA*_*1*,_ and *HS* to a predefined value. The MDH model (SEM or GSEM) does not converge with STATA. With these problems of convergence, GSEM, was always better than SEM (ΔAIC = 433.4 and ΔBIC = 436.2). On the contrary, the FCH converges using both SEM and GSEM. Even for FCH, GSEM provided a better fit than SEM (ΔAIC = 438.9, ΔBIC = 441.7). In synthesis, this analysis shows that FCH is always preferred to MDH by having lower AIC and BIC values both when fitted using SEM and GSEM (ΔAIC and ΔBIC >140 always). Due to these results, the MDH model will not be considered in the following analyses. Path coefficients for GSEM-FCH models are shown in Table 3. All coefficients are highly significant (P<0.003). Noteworthy, the path coefficient for ASS_T_ is positive and not negative as expected.

**Table 3:** Standardized path coefficients, SE, and p-value for FCH in GSEM.

Variables	Path coefficients	GSEM	
		Estimate ± SE	P
*Mating success (η*_*2*_*)*			
*HS*	*λ*_*5*_	0.630 ± 0.065	<0.001
*CourtS*	*λ*_*6*_	0.896 ± 0.019	<0.001
*CopS*	*λ*_*7*_	2.387 ± 0.138	<0.001
*Lek attendance (η*_*1*_*)*			
*LA*_*1*_	*λ*_*3*_	0.936 ± 0.035	<0.001
*LA*_*2*_	*λ*_*4*_	0.969 ± 0.038	<0.001
*Antler shape (ξ*_*1*_*)*			
*ASS*_*T*_	*λ*_*1*_	0.480± 0.038	<0.001
*TotS*	*λ*_*2*_	0.379 ± 0.121	0.002
*η*_*1*_ *on ξ*_*1*_	*γ*_*1*_	0.585 ± 0.194	0.003
*η*_*2*_ *on η*_*1*_	*β*_*1*_	0.330 ± 0.093	<0.001

Variables and symbols are detailed in the text.

### Model comparisons

The comparison of the models is reported in Table 4. It clearly appears that the precision of MAM models for LMs and GLMs is higher than that of the corresponding full models. Considering the median CV values, the two less precise models are GLM_4_ (median CV = 1.089) and LM_1_ (median CV = 0.828), while the more precise models are GLM_4,r_ (median CV = 0.162) and GLM_2,r_ (median CV = 0.148). LMs and GLMs were clearly outperformed by both the SEM (median CV = 0.079) and, to a larger extent, by GSEM (median CV = 0.059), whose coefficient CV values range from 0.02 to 0.319.

**Table 4:** Summary results of LM, GLM, SEM, and GSEM.

*Model*	*K*_*1*_	*ASS*_*T*_	*TotS*	*Dom*	*Ds*	*LA*_*1*_	*LA*_*2*_	*HS*	*CourtS*	*Median*
*LM*_*1*_	2	1.883	1.007	0.645	0.859	0.798	1.600	0.182	0.339	0.828
*LM*_*1*,*r*_	2							0.171	0.286	0.228
*GLM*_*1*_	5	0.624	0.227	8719	0.365	0.369	1.177	0.098	0.103	0.367
*GLM*_*1*,*r*_	5		0.235		0.262	0.214		0.086	0.097	0.214
*GLM*_*2*_	2	4.569	0.783	3.369	1.299	0.613	2.033	0.199	0.132	1.041
*GLM*_*2*,*r*_	2							0.178	0.119	0.148
*GLM*_*3*_	5	0.625	0.227	2311	0.335	0.368	1.176	0.098	0.103	0.351
*GLM*_*3*,*r*_	5		0.235		0.262	0.214		0.086	0.097	0.214
*GLM*_*4*_	2	4.946	0.838	3.395	1.341	0.644	2.204	0.219	0.144	1.089
*GLM*_*4*,*r*_	2							0.193	0.132	0.162
*GLM*_*5*_	3	0.508	0.296	0.826	2.75	0.605	100	0.128	0.886	0.715
*GLM*_*5*,*r*_	3	0.456	0.520					0.097		0.456
*SEM-FCH*	7	0.079	0.312			0.051	0.046	0.143	0.131	0.079
*GSEM-FCH*	7	0.079	0.319			0.037	0.039	0.103	0.021	0.059

On the left: type of model, number of significant (P<0.05) coefficients. On the right: coefficient of variation (CV) of regression parameters and their median. MAMs are denoted by the suffix r. Variable names are detailed in the text. All models have the same numbers of observations (N = 118). K_1_ is the number of significant regression coefficients.

**Fig 3:**
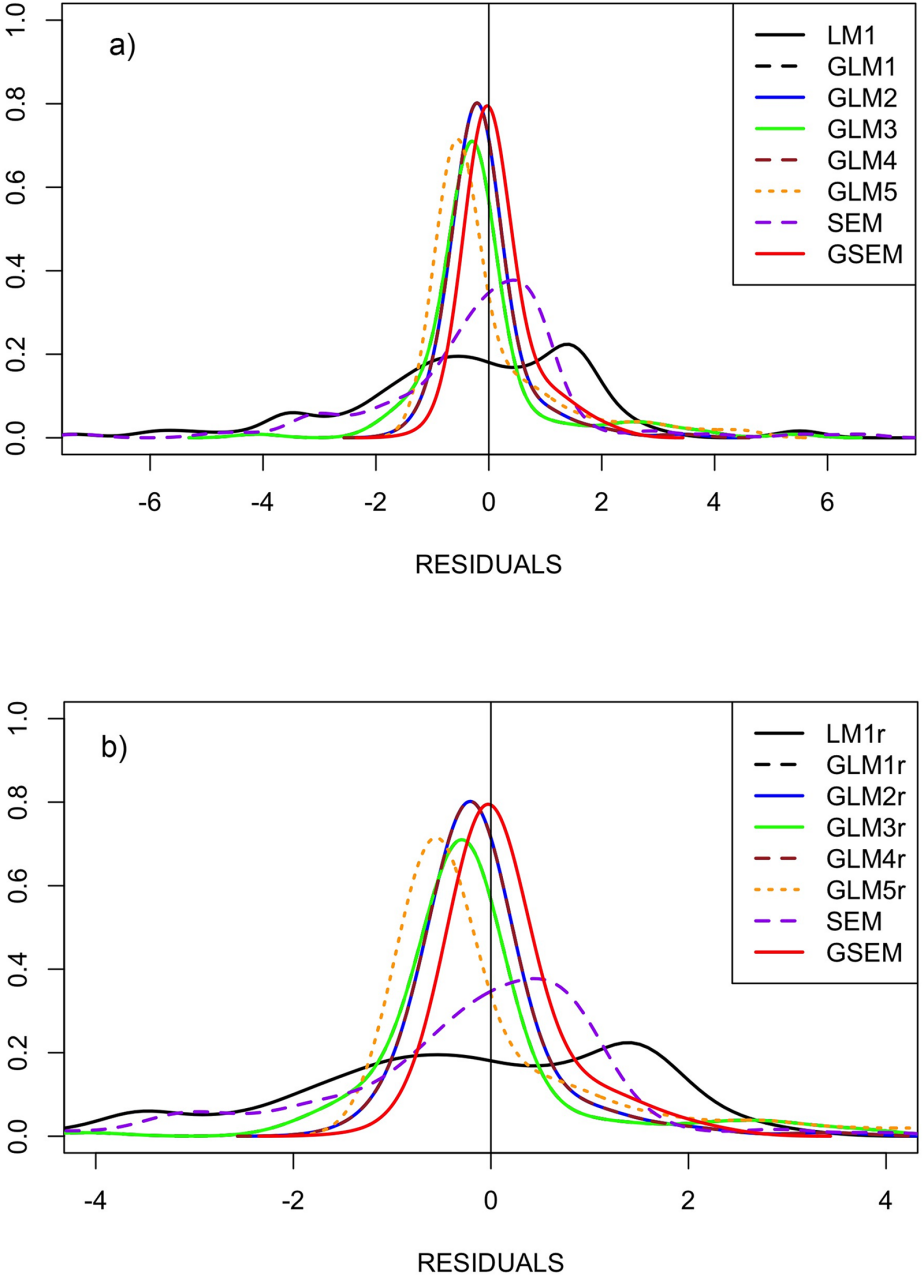
Model validation graph. **a) Distribution of standardized residuals of GLMs, SEM, and GSEM models. For LMs and GLMs, both full (a) and reduced models (b) are shown.** Models are in Table 1. The respective descriptive statistics of the different distribution models considered in this paper are reported in Table 5.

Comparable results are obtained when analysing the distribution of residuals (Table 5, Fig 3). In LMs, the variance is very large, and the distribution is strongly leptokurtic with heavy tails (Fig 3). As a comparison, statistics of the distribution of residuals for LMs with transformed response variables are shown in S6 Table. These distributions are characterised by large variances and kurtosis, and none is centred on zero.

**Table 5:** Mean, variance, and kurtosis for residual distributions of the different models considered in this paper.

*Model*	*Mean*	*Var*	*Kurtosis*
*LM*_*1*_	0	13.92	29.96
*LM*_*1*,*r*_	0	14.78	30.58
*GLM*_*1*_	-0.26	1.18	9.23
*GLM*_*1*,*r*_	-0.27	1.2	9.32
*GLM*_*2*_	-0.21	0.32	9.45
*GLM*_*2*,*r*_	-0.23	0.33	8.30
*GLM*_*3*_	-0.10	1.32	9.86
*GLM*_*3*,*r*_	-0.11	1.30	9.44
*GLM*_*4*_	-0.08	0.36	14.44
*GLM*_*4*,*r*_	-0.09	0.38	18.41
*GLM*_*5*_	0.01	1.28	7.55
*GLM*_*5*,*r*_	0.01	1.37	7.22
*SEM—FCH*	0	6.10	32.56
*GSEM—FCH*	0.14	0.28	6.84

Models are in Table 1.

GLMs perform better than LMs (Table 5), distributions remain leptokurtic, but variances are smaller, and the mean is slightly biased low (Fig 3a and 3b). The residuals associated with SEM-FCH (actually to the relationship between *CopS* and *η*_*2*_), although their mean is close to 0, have a strongly leptokurtic distribution and have a variance much larger than that of GLMs (but not LMs). Finally, the residuals associated with GSEM-FCH have a low variance and the least value of kurtosis among the studied models.

Interestingly the number of regression coefficients that are significant is maximal in SEM and GSEM (Table 4). Since results indicate that GSEM-FCH is the model more appropriate for our data (lower AIC/BIC, lower residuals’ variance, and lower CV median), it is interesting to investigate total effects (cfr. S3 Text) for this model (Table 6). Noteworthy, the impact of *ξ*_*1*_ and *η*_*1*_ on CopS is of similar size with respect to *η*_*2*_, while *ξ*_1_ and *η*_*1*_ have much smaller effects on *CourtS* or *HS* than *η*_*2*_, which suggests a remote causation for *CopS*. The impact of *ξ*_*1*_ on both *ASS*_*T*_ and *TotS*, but to different degree, is more relevant for *ASS*_*T*_ than *TotS*.

**Table 6:** Total effects of GSEM in FCH model.

**Manifest Variables**		**GSEM**	
	*ξ*_*1*_	*η*_*1*_	*η*_*2*_
*ASS*_*T*_	0.480		
*TotS*	0.379		
*LA*_*1*_	0.548	0.936	
*LA*_*2*_	0.567	0.969	
*HS*	0.122	0.208	0.630
*CourtS*	0.173	0.296	0.896
*CopS*	0.193	0.330	2.387
**Latent Variables**			
*ξ*_*1*_		0.585	0.193
*η*_*1*_			0.330

## Discussion

The data collected at Castelporziano on the mating behaviour of fallow bucks represents a typical example of the many studies performed on the leks of this species [11, 12, 34, 23] and other species of vertebrates [9, 12, 29]. These behavioural studies are important not only to identify the proximate causes of mate selection, but also for determining the intensity of sexual selection and understanding the evolution of exaggerated traits in males.

A literature review (cfr. S2 Text and S2 Table) allowed us to select the more popular methods used in previous research and to contrast them with innovative GSEMs. The use of the same dataset to compare different statistical methodologies is useful for evaluating their relative efficiency in data fitting. In general, LMs appear to be severely biased, and although GLMs may improve the reliability of the results, they overlook several important effects and the estimated coefficients still have low precision, which severely jeopardizes their predictive capacity. It is worth stressing that data transformation is not appropriate to normalize data distribution, since results appear extremely sensitive to the specific function used. This problem is exacerbated by the large number of zeros in the distribution of male copulatory success.

The introduction of GSEMs in the analysis of lek mating appears to represent a relevant leap ahead in the field. Our study provided evidence of several advantages of GSEMs compared to GLMs. First, the collinearity of predictors is no longer a nuisance provided that an appropriate measurement model is built, so we save part of the information collected in the field, which is usually lost in GLMs to reduce variance inflation [36]. Second, GSEMs are a flexible tool since they allow contrasting different casual models (e.g. using AIC, BIC, or other fit indexes) which must be formulated *a–priori*. In comparison to both LM and GLM, a proactive model formulation improves the awareness of the biological significance of the mechanism to be tested and allows scholars to modify a basic theoretical construct by introducing specific paths which are known or thought to be relevant in each particular study condition. This feature of SEMs allows us to include both general theoretical statements and specific conditions in the same model, which are then evaluated together. The publication of the variance-covariance matrix has the advantage of allowing other scholars to replicate the results easily and to propose different theoretical models pertinent to the system of interest, and in doing so, improve the transparency of the research and the full reproducibility of the results. However the availability of rough data can be useful to adjust the standard errors. Finally, SEM/GSEM help to control for measurement errors, a much neglected flaw in most quantitative analyses.

GSEM represents a bridge between the descriptive approach developed in LM and GLM and experimental tests with manipulative treatments; indeed the consistency of alternative causal paths can be tested, and when possible, the results can be used to develop more stringent experiments.

The importance of using GSEMs is well represented by the between-method comparisons reported in this study. First, we were able to show that, with respect to GLMs and even more to LMs, GSEMs suggest the potential influence of a larger number of predictors, in other words more informative models can be developed. This may have a strong impact on the interpretation of the study. For instance, both LMs and GLMs (except for the Poisson models) were unable to detect any effect of predictors referring to male dominance, which are however present, albeit with a small effect. Indeed in the literature, several authors were unable to detect these effects at all (e.g. [8,10, 14, 73]).

The second relevant aspect of GSEMs is the increased precision of the estimates of the regression coefficients. For several predictors GLMs yielded CV values >50% which are clearly unacceptable, while with GSEMs, CVs were often <10%, a precision we consider “acceptable” for a field study. The analysis of residuals in GSEMs and GLMs confirmed that the former allowed a better fitting of the data than the latter.

While these results are not meant to disprove the available results about lek breeding of fallow deer based on linear models, the analysis of our dataset illustrates some advantages in using GSEMs for discrete responses. SEMs are more flexible and have more parameters than GLMs and may better fit the data of interest. Indeed, the formal definition of contrasting working hypotheses, such as FCH and MDH in this study, is illustrative of the potentiality of SEM for hypotheses testing. On the other hand, with respect to LMs and GLMs, SEM are data hungry and Shipley [41] gives a rule of thumb to decide the number of parameters that can be safely estimated given a certain sample size.

The practical use of GSEM presents several difficulties. The main problem is that the likelihood of SEMs with latent variables is generally multimodal, and there is a need for a general algorithm to locate the global maximum. Moreover, the algorithm sometimes does not converge to a proper solution and this usually suggests that the model is not identifiable (at least in some parts). A partial remedy is to include reasonable identifiability constraints. In path analysis or with GLMs, the problems of non-convergence are generally absent.

One drawback that may limit a wider diffusion of GSEM is that the possibility of modelling non-normal variables is not yet implemented in widespread statistical packages, such as SAS, R, or S-plus. In this paper, GSEMs have been implemented in Mplus and STATA. We support the importance of using both packages, because they present complementary advantages and disadvantages. For instance STATA provides case-specific residuals, which are not outputted by Mplus, but Mplus returns the standardized path coefficients and total, direct, and indirect effects which STATA does not compute. The use of Mplus requires caution, because to get convergence, it automatically constrains the value of some path coefficients to be one. In STATA, constraints have to be specifically applied, which is a feature that improves awareness for the user. In our experience, STATA is much slower than Mplus, but it is well-documented; in some cases STATA, unlike Mplus, failed to converge (e.g. with MDH). However, STATA implements only a limited GSEM procedure, for example it does not support ZIP or ZINB distributions despite the greater flexibility in model specification.

The analyses in this paper were developed under a frequentist approach. A Bayesian analysis of our data with GSEM is outside the scope of the present study and would require further research especially as far as the choice of priors is concerned. For an introduction to Bayesian SEMs see Kaplan & Depaoli [74].

The importance of this study lies in the fact that, to our knowledge, it is the first comparative study of SEM and GSEM models. We believe that past work should be reviewed in the light of the results obtained here. Specifically, the results from studies using LMs should be considered with great caution, particularly in those cases where assumptions were clearly violated and transformations to normalise non-normal variables were applied. Interestingly, Grace et al. [75] analysed the species richness-productivity relationships using SEM and showed that an integrative model has an higher explanatory power than traditional linear models, since SEM allows us to integrate competing hypothesis into a single model. Furthermore, SEMs help to solve the Simpson’s paradox [47]. Finally, it is important to stress that the use of GSEMs can be extended to other behavioural and ecological contexts characterised by non-normal distributions of variables. SEMs are getting traction in behavioural studies and in ecology. According to the WOS (accessed on the 13/5/2016), the number of ecological and zoological papers using SEM is increasing by 7% per year. Thus, GSEM can find wider and wider opportunities for application. In particular, the possibility of using SEMs to test hypotheses in competition and investigate both remote and proximate effects is of particular interest in ecological and evolutionary studies. The present study can therefore stimulate the application of GSEM to different study cases.

## Supporting information

S1 Text Details on data, validation and measures computation. (DOCX)

S2 Text A review of pertinent papers about lek in mammals and bird. (DOCX)

S3 Text A description of SEMs modelling including the total effects. (DOCX)

S4 Text Mplus and STATA codes used to generate SEM and GSEM. (DOCX)

S5 Text Maximum likelihood estimates from the log and root square transformed model including R code. (DOCX)

S6 Text Template of data set. (DOCX)

S1 Table Table with variables name. (DOCX)

S2 Table Table of papers review about lek in mammals and bird. (DOCX)

S3 Table Table with the complete list of variables name of the models and the respective path coefficients. (DOCX)

S4 Table GLMs parameters estimates. (DOCX)

S5 Table Variance-covariance and correlation matrix used in SEM and GSEM for FCH and MDH models. (DOCX)

S6 Table The results of a comparison of residuals statistical analysis for LMs with transformed response variable. (DOCX)

S1 Fig Study area in Castelporziano (Rome, Italy). (PDF)

S2 Fig Phenological characters of fallow deer buck (*Dama dama*) in Castelporziano (Rome, Italy). (PDF)

S3 Fig An example of SEM. (PDF)

S1 Dataset Data set to implement SEM and GSEM with STATA and Mplus. (XLSX)
