# Supplementary material for: Generalized structural equations improve sexual-selection analyses
Source: PLoS One. 2017 Aug 15;12(8):e0181305. doi: 10.1371/journal.pone.0181305 (PMC5557364; doi:10.1371/journal.pone.0181305)
Supplement: S1 Text — (DOCX) [file pone.0181305.s001.docx]

**S1 Text**

**Details on data validation and measures computation**

Adult bucks were individually identified using a database of antler morphology. Fallow deer were videotaped from a high seat at the margin of the lek area, and pictures of antlers were used to estimate the number of small (*SS*) and large (*LS*) spellers, according to Pelabon & Joly (2000). All antlers missing tines, without palms, or were broken were excluded from the analysis. Two variables were used: a) the total number of spellers (*TotS*) and b) a measure of fluctuating asymmetry for small spellers (Møller 1990; Møller *et al.* 1996), computed as:

*ASS_T_ = |SS_r_ - SS_l_ |*,

where *SS_r_* and *SS_l_* are the number of small spellers on the right and the left antler, respectively.

Animals were systematically observed from dawn to dusk (38 days, 396 hours in 1991, 40 days, 337 hours in 1992) at hourly intervals, and the position of each identified buck in the lek, its activity, and the number of females and fawns within its territory were recorded. All fights and copulations were also noted. A total of 695 fights were observed in 1991 and 186 in 1992. Only the fights which ended with the victory of one of the rivals were considered.

To validate the estimates of the number of large (*LS*) and small (*SS*) spellers, we used a set of 69 cast antlers, collected at Castelporziano in the period 1991-1998, for which pictures drawn while animals were in the lek were also available. One operator evaluated the number of large and small spellers from drawings, while another independently obtained the same information from photos of the casted antlers. The results showed that the two estimates of the number of spellers were consistent (Pearson’s correlation; *SS*: *r*=0.57, *N*=23, *P*=0.004; *LS, r*=0.67*, N*=23*, P*=0.005). When casted antlers were found, a complete set of biometric measurements were also taken, including their weight (*AW*).

**References**

Møller, A. P. (1990) Fluctuating Asymmetry in male sexual ornaments may reliably reveal male quality. *Animal Behaviour*, 40*,* 1185-1187.

Møller, A. P, Saler, J. J. & Zamora–Munoz, C. (1996) Horn asymmetry and fitness in gemsbok, *Orix g. gazzella. Behavioural Ecology*, 3, 247- 253.

Pélabon, C. & Joly, P. 2000. What, if anything, does visual asymmetry in fallow deer antlers reveal? *Animal Behaviour*, 59, 193-199.
