# Supplementary material for: Generalized structural equations improve sexual-selection analyses
Source: PLoS One. 2017 Aug 15;12(8):e0181305. doi: 10.1371/journal.pone.0181305 (PMC5557364; doi:10.1371/journal.pone.0181305)
Supplement: S2 Text — (DOCX) [file pone.0181305.s002.docx]

**S2 Text**

**Literature review**

We reviewed the main statistical methods used, pinpointing their weaknesses and the solutions adopted. The choice of paper included in the meta-analysis was carried out by searching on the Web of Science^TM^ in on February 2016 for the 1980-2016 period, using as keywords: lek (AND deer OR antelope OR mammal) AND (mating success OR copulatory success). The search found 109 papers among which we selected those whose working hypotheses were comparable with those of our paper. We analyzed a total of 31 papers, listed in S2 Table.

In the majority of cases (n=15), some type of multiple regression was used. There were three cases where authors only presented descriptive statistical analyses, in three other cases chi-square or Fisher's tests were used, three papers used simulations and ten other studies employed Pearson’s or Spearman’s rank correlations, without any attempt to control for spurious correlations.

In eleven of the papers that presented a linear (LM) or a linear mixed-effects model (LMM), the response variable (the number of copulations achieved by each male) was used as is (1) or log/square-root transformed. One author used a logistic regression model, after transforming copulation numbers into a binary variable (present/absent). Five papers employed GLM (or GLMM) explicitly using non-normal distributions (Poisson or Zero Inflated Poisson, ZIP) for the response variable. In one case the variable was normalized using Blom’s score. Finally, only one paper used path analysis with manifest variables, but with a violation of normality assumption of the response variable. In this case, the authors used a transformation of the original data based on Blom’s scores, a rank procedure that yields normally-distributed variables.
