# Supplementary material for: Generalized structural equations improve sexual-selection analyses
Source: PLoS One. 2017 Aug 15;12(8):e0181305. doi: 10.1371/journal.pone.0181305 (PMC5557364; doi:10.1371/journal.pone.0181305)
Supplement: S3 Text — (DOCX) [file pone.0181305.s003.docx]

S3 Text

Structural Equations Model (SEM) and Generalized Structural Equation Model (GSEM)

A detailed presentation of structural equations may be found in Shipley (2000). The development of a structural model requires the *a-priori* definition of links among model variables in the form of a regression equations system. The goal of this class of models is minimize the difference between estimates and expectations variance-covariance matrix of data.

We assume causal relationships going from the latent variable to the manifest variables in its block. Latent variables are unobserved factors denoted, *η_1_, η _2_, η _n_,* that represent an hypothetical construct that can be inferred by the way it influences manifest or observed variables (continuous, *Y*_i_=*y_1_, y_2_,..,y_n_)* (Shipley 2000*;* Pugesec 2003; Muthén& Asparouhov 2015).

A SEM model is composed by two sub-models: a measurement model that describes the relationships between latent variables and their manifest variables and a structural or causal model that constitutes a directional chain system that describes the relationship between the constructs of theoretical interest (latent variables) using *path diagrams* (S3 *Fig*).

Structural coefficients (*γ, β*) represent the effects of each independent variable on the dependent variable.

A manifest variable, in a SEM with latent variables, plays a role of endogenous variable if it is predicted by another variable in the model and is therefore a response variable; it is assumed to be generated as a linear function of its latent dimension and the residual error term represents the imprecision in the measurement process. An exogenous variable whose variation is not explained in a model (i.e. antler shape and FA of small spellers).

In synthesis (Bollen, 1989; Chen *et al.* 2001; Kolenikow & Bollen, 2012) a SEM of a LISREL type is composed by a system of three equations given by:

* η = a _η_ +* ***Β****η *+* ****Г***ξ + *ζ*

*x =*  *a _x_ * + ***Λ****_x_ξ* +* δ* (*1S3 eqns*)

*y = a _y_* **+*****Λ****_y_ η* + *ε *

The first equation represents the causal model, x and y represent the measurement model. The standard assumptions are made that ξ*  ζ  δ  ε *are uncorrelated, and have covariance matrices *Φ, y_ζ _y_δ_ y_ε_ *Based on the SEM model the implied covariance matrix of *p* = (*x’, y’*)*’* is given by $\sum(\theta)$where the parameter vector *j*contains the intercept vectors* a _x_ a _y_* The ***B*** and ***Г*** matrices are the regression parameters required to be estimated. The ***Λ****_x_* and ***Λ****_y_* are the factor loadings for *η* and *ξ*. The *ε* and *δ* vectors are the measurement errors for *y* and *x* respectively and *ζ* is referred to the disturbance.

The researcher is interested in parameter estimates of the coefficients and the overall goodness of fit test of the model:

$H_{0} :COV\left[ p \right]= \sum(\theta)$

* *$H_{1} :\forall\theta COV\left[ p \right]\neq\sum(\theta)$* *

* *

with dimension **$\pi*= \pi(\pi+1)/2$**

For instance, in the following equation:

*y_4_* = *λ_1_y_1_* + *λ_2_y_2_* +*…..*+ *ε _y4_*,

*y_4_* represents the dependent variable,*y_1_, y_2_*,.., etc. the independent variables and *ε_y4_* the error term for *y_4_*; *λ_1_*, *λ_2_* are the parameters to be estimated. Given a set of dependent variables *y_1_, y_2_*, ecc… our aim is to minimise the difference between experimental and predicted variance-covariance matrices $(COV \left[ p \right]= \sum(\theta))$

The fundamental hypothesis underlying these approaches is that the implied covariance matrix of the manifest variables is a function of the model parameters.

SEM are related to factorial analysis where underlying and unknown factors are evidenced by the correlations among the manifest variables. On the other side a modification in the structural part implies a difference about the theoretical hypothesis to be tested.

In a structural equation, standardized parameters *λ_1_*, *λ_2_*.., represent the effects of each independent variable on the dependent variable. Beside such direct effects we can also compute indirect effects. Consider the system of structural equations:

*y_3_* = *λ*_1_*y_1_* + *λ*_2_ *y_2_* + *ε* *_y3_*,

*y_4_* = *λ*_3_*y_3_* + *ε* *_y4_*.

It is clear that *y_3_* has a direct effect on *y_4_* but *y_1_* may also indirectly influence *y_4_* via *y_3_* with intensity *λ*_1_*λ*_3_. Our aim is to evaluate total effects (direct and indirect) of manifest variables on copulatory success calculated by multiplication structural coefficients (total effect= direct effect + indirect effect).

SEM can test different working hypothesis, by comparing alternative models. A straightforward method to evaluate model fitting is to inspect the distribution of standardised residuals. Parameter estimation is performed by maximum likelihood (ML) estimation or maximum likelihood robust (MLR) estimation. The unknown parameters of the model are estimated so as to make the variances and covariances that are reproduced from the model in some sense close to the observed data. A good model would allow very close approximation to the data. The literature is full of discussion about the opportunity of using goodness-of-fit indexes (see Shipley 2000:188-194).

Since we compared non-nested models with identical number of variables and sample size, we adopted the AIC (Akaike, 1974).

SEM with Generalized response (GSEM) use a generalized measurement part with ordered categorical and continuous variables, grouped together also in the same latent construct, to emphasize the nature of the Poissonian process (ZIP) or Negative Binomial process (ZINB), that produce count variables like the number of success, the rate of courtship, the number of female and finally copulatory success (Muthén & Muthén, 2015, Mplus, 7.4 release*)*.

For instance, a model with two latent variables, two exogenous and two endogenous manifest variables described by the following system of equations:

*η *_1_* = γξ*_1_* + ζ*_1_

*x*_1_ =*λ*_1_ *ξ *_1_ + *δ_1_*

*x_2_ =λ*_2_ *ξ *_1_ *+ δ *_2_(*2S3 eqns*)

*y_1_ =λ*_3_ *η *_1_ + *ε *_1_

*y_2_* =*λ*_4_ *η *_1_ + *ε *_2_

can be graphically represented by the path diagram in S3 Fig. Variable *x_1_* and *x_2_* are manifest exogenous variables because they are not explained by the model, while *y_1_* and *y_2_* depend on the structure of the model and are called manifest endogenous variables. We denote by *ξ_1_* the latent exogenous variables, and by *η*_1_ the latent endogenous variable. Variables *δ_1_*, *δ_2_*, *ε_1_*, *ε_2_* and *ζ_1_*are typically assumed normally-distributed error terms while *γ, λ_1_, λ_2_, λ_3_* and *λ_4_* are the path coefficients to be estimated. The variance of latent variables was set equal to 1.

***References***

Akaike, H. (1974) A new look at the statistical model identification. *IEEE Transactions on Automatic Control*, 716–723.

Bollen, K.A. (1989) Structural equations with latent variables. *New York, John Wiley & Sons*.

Chen, F., Bollen, K. A., Paxton, P., Curran, P., & Kirby, J. (2001). Improper solutions in structural equation models: Causes, consequences, and strategies. *Sociological Methods and Research*, *29*, 468-508.

Kolenikov, S. & Bollen, K. A. (2012). Testing negative error variances: Is a Heywood case a symptom of misspecification? *Sociological Methods and Research*, *41*, 124-167.

Pugesek, B.H., Tomer, A., Eye, A. (2003) Structural equation modeling. *Cambridge University Press, Cambridge.*

Muthén, B.& Asparouhov, T. **(**2015) A general structural equation model with dichotomus, ordered categorical, and continuous latent variable indicators. *Psychometrika*, 49, 115-132.

Muthén, B.& Muthén, L. (2015)Mplus 7 ver. 7.4 Copyright © 2012-2015.

Shipley, B. (2000) Cause and correlation in biology: a user’s guide to path analysis, structural equations and causal inference. *Cambridge University Press*.
