# Supplementary material for: Generalized structural equations improve sexual-selection analyses
Source: PLoS One. 2017 Aug 15;12(8):e0181305. doi: 10.1371/journal.pone.0181305 (PMC5557364; doi:10.1371/journal.pone.0181305)
Supplement: S4 Text — (DOCX) [file pone.0181305.s004.docx]

**S4 Text**

**STATA CODE**

**1. SEM INSTRUCTIONS FOR FEMALE CHOICE MODEL**

clear

use "/Users/Desktop/GSEM/datiSEM.dta"

set more off

sem (xi@1 -> asst, ) (xi -> tots, ) (xi -> eta1, ) (eta1 -> la1, ) (eta1 -> la2, ) (eta1 -> eta2, ) (eta2 -> hst, ) (eta2 -> cops, ) (eta2 -> courts, ), vce(robust) latent(xi eta1 eta2 ) cov( xi@1 e.eta1@1 e.eta2@1) nocapslatent

predict ch hh oh, xb(cops hst courts)

predict e1h e2h, xblatent(eta1 eta2)

gen res = cops - ch

summarize res

**2. SEM INSTRUCTIONS FOR DOMINANCE MALE MODEL**

use "/Users/Desktop/GSEM/datiSEM.dta"

sem (xi@1 -> dom, ) (xi -> ds, ) (xi -> eta1, ) (eta1@1 -> la1, ) (eta1 -> la2, ) (eta1 -> eta2, ) (eta2@1-> hst, ) (eta2 -> cops, ) (eta2 -> courts, ), vce(robust) difficult latent(xi eta1 eta2 ) cov( xi@1 e.eta1@1 e.eta2@1) nocapslatent

**3. GSEM INSTRUCTIONS FOR FEMALE CHOICE MODEL**

clear

use "/Users/Desktop/GSEM/datiSEM.dta"

set more off

gsem (xi@1 -> asst, ) (xi -> tots, ) (xi -> eta1, ) (eta1 -> la1, ) (eta1 -> la2, ) (eta1 -> eta2, ) (eta2 -> hst, ) (eta2 -> cops, family(poisson) link(log)) (eta2 -> courts, ), difficult latent(xi eta1 eta2 ) cov( xi@1 e.eta1@1 e.eta2@1) nocapslatent

predict coppa, mu outcome(cops)

predict latta, latent(eta2)

gen rr = cops - coppa

summarize rr

**4. GSEM INSTRUCTIONS FOR DOMINANCE MALE MODEL**

use "/Users/Desktop/GSEM/datiSEM.dta"

gsem (xi@1 -> dom, ) (xi -> ds, ) (xi -> eta1, ) (eta1@1 -> la1, ) (eta1 -> la2, ) (eta1 -> eta2, ) (eta2@1-> hst, ) (eta2 -> cops, family(poisson) link(log)) (eta2 -> courts, ), vce(robust) intmethod(ghermite) difficult latent(xi eta1 eta2 ) cov( xi@1 e.eta1@1 e.eta2@1) nocapslatent

predict copsa, mu outcome(cops)

predict latsa, latent(eta2)

gen rrm = cops – copsa

summarize rrm

**MPLUS CODE**

**5 SEM INSTRUCTIONS FOR FEMALE CHOICE MODEL**

TITLE: SEM with latent variables

ASST y1

TotS y2

LA1 y3

LA2 y4

dom y5

ds y6

HST y7

HS y8

CopS y9

CourtS y11;

DATA:

FILE = C:/Users/Desktop/Mplus/datiSEM1.csv;

VARIABLE:

NAMES = y1-y8 u9-u10 y11;

USEVARIABLES= y1 y2 y3 y4 y7 u9 y11;

ANALYSIS:

TYPE=GENERAL;

ESTIMATOR=MLR;

STARTS=5;

MODEL:

!* The metric of the factor is defined by fixing the factor variance at 1*!

f3 by y7* u9 y11;

f3@1;

f2 by y3* y4;

f2@1;

f1 by y1 y2;

f1@1;

f2 ON f1;

f3 ON f2;

MODEL INDIRECT:

y1 IND f1;

y2 IND f1;

y3 IND f2;

y4 IND f2;

y7 IND f3;

u9 IND f3;

y11 IND f3;

y7 IND f1;

u9 IND f1;

y11 IND f1;

y7 IND f2;

u9 IND f2;

y11 IND f2;

y3 IND f1;

y4 IND f1;

f2 IND f1;

f3 IND f2;

f3 IND f1;

OUTPUT: TECH1 TECH3 TECH4

SAMPSTAT STANDARDIZED RESIDUAL CINTERVAL;

SAVEDATA:

FILE IS DATISEMFCH.TXT;

PLOT: TYPE=PLOT3;

**6 SEM INSTRUCTIONS FOR DOMINANCE MALE MODEL**

TITLE: SEM with latent variables

DATA:

FILE = C:/Users/Desktop/Mplus/datiSEM1.csv;

VARIABLE:

NAMES = y1-y8 u9-u10 y11;

USEVARIABLES= y3 y4 y5 y6 y7 u9 y11;

ANALYSIS:

TYPE=GENERAL;

ESTIMATOR=MLR;

STARTS=5;

MODEL:

!* The metric of the factor is defined by fixing the factor variance at 1*!

f3 by y7 u9 y11;

f3@1;

f2 by y3 y4;

f2@1;

f1a by y5 y6;

f1a@1;

f2 ON f1a;

f3 ON f2;

MODEL INDIRECT:

y5 IND f1a;

y6 IND f1a;

y3 IND f2;

y4 IND f2;

y7 IND f3;

u9 IND f3;

y11 IND f3;

y7 IND f1a;

u9 IND f1a;

y11 IND f1a;

y7 IND f2;

u9 IND f2;

y11 IND f2;

y3 IND f1a;

y4 IND f1a;

f2 IND f1a;

f3 IND f2;

f3 IND f1a;

OUTPUT: TECH1 TECH3 TECH4

SAMPSTAT STANDARDIZED RESIDUAL CINTERVAL;

SAVEDATA:

FILE IS DATISEMMDH.TXT;

PLOT: TYPE=PLOT3;

**7 GSEM INSTRUCTIONS FOR FEMALE CHOICE MODEL**

TITLE: GSEM with latent variables

DATA:

FILE = C:/Users/Desktop/Mplus/datiSEM1.csv;

VARIABLE:

NAMES = y1-y8 u9-u10 y11;

USEVARIABLES= y1 y2 y3 y4 y7 u9 y11;

COUNT= u9 (p);

ANALYSIS:

INTEGRATION=30;

TYPE=GENERAL;

MCONVERGENCE=0.01;

ESTIMATOR=MLR;

STARTS=5;

MODEL:

!* Measurement Model*!

f3 by y7* u9 y11;

f3@1; !* The metric of the factor is defined by fixing the factor variance at 1*!

f2 by y3* y4;

f2@1;

f1 by y1 y2;

f1@1;

!* Structural Model*!

f2 ON f1;

f3 ON f2;

MODEL INDIRECT: !* Total, direct and indirect causal effects*!

y1 IND f1;

y2 IND f1;

y3 IND f2;

y4 IND f2;

y7 IND f3;

u9 IND f3;

y11 IND f3;

y7 IND f1;

u9 IND f1;

y11 IND f1;

y7 IND f2;

u9 IND f2;

y11 IND f2;

y3 IND f1;

y4 IND f1;

f2 IND f1;

f3 IND f2;

f3 IND f1;

OUTPUT: TECH1 TECH3 TECH8 TECH4 TECH10

SAMPSTAT STANDARDIZED RESIDUAL CINTERVAL;

SAVEDATA:

FILE IS DATIGSEMFCH.TXT;

PLOT: TYPE=PLOT3;

**8 GSEM INSTRUCTIONS FOR DOMINANCE MALE MODEL**

TITLE: GSEM with latent variables

DATA:

FILE = C:/Users/Desktop/Mplus/datiSEM1.csv;

VARIABLE:

NAMES = y1-y8 u9-u10 y11;

USEVARIABLES= y3 y4 y5 y6 y7 u9 y11;

COUNT= u9 (p);

ANALYSIS:

INTEGRATION=30;

TYPE=GENERAL;

MCONVERGENCE=0.01;

ESTIMATOR=MLR;

STARTS=5;

MODEL:

f3 by y7 u9 y11;

f3@1; !* The metric of the factor is defined by fixing the factor variance at 1*!

f2 by y3 y4;

f2@1;

f1a by y5 y6 ;

f1a@1;

f2 ON f1a;

f3 ON f2;

MODEL INDIRECT:

y5 IND f1a;

y6 IND f1a;

y3 IND f2;

y4 IND f2;

y7 IND f3;

u9 IND f3;

y11 IND f3;

y7 IND f1a;

u9 IND f1a;

y11 IND f1a;

y7 IND f2;

u9 IND f2;

y11 IND f2;

y3 IND f1a;

y4 IND f1a;

f2 IND f1a;

f3 IND f2;

f3 IND f1a;

OUTPUT: TECH1 TECH3 TECH8 TECH4 TECH10

SAMPSTAT STANDARDIZED RESIDUAL CINTERVAL;

SAVEDATA:

FILE IS DATIGSEMMDH.TXT;

PLOT: TYPE=PLOT3;
