# Supplementary material for: Generalized structural equations improve sexual-selection analyses
Source: PLoS One. 2017 Aug 15;12(8):e0181305. doi: 10.1371/journal.pone.0181305 (PMC5557364; doi:10.1371/journal.pone.0181305)
Supplement: S5 Text — (DOCX) [file pone.0181305.s005.docx]

**S5 Text**

**Log-Likelihood of discrete and continuous distributions to calculate AIC, BIC and R code**

The Information criterion AIC or BIC provides a way to compare nested and non-nested models. The AIC is computed using the formula AIC=-2log(L)+2q, while BIC = -2log(L)+qlog(n) where L is the likelihood, q the number of estimated parameters in the model and n is the number of observations. The log-likelihoods obtained from a model fitted using a discrete or a transformed response variables are, however, not comparable. A solution is provided by the formula suggest by Weiss (2010), which allows to compare models using an untrasformed and a log-transformed response variable:

$$L\left( y \right)=\prod_{i=1}^{n} dnorm(logy_{i}; \mu_{i}, \sigma)\frac{1}{y_{i}}$$

or in terms of log-likelihood:

$$logL\left( y \right)=\sum_{i=1}^{n} log\left[ dnorm(logy_{i};\mu_{i} ;\sigma)\frac{1}{y_{i}} \right]$$

or in terms of log-likelihood for square root transformed response model:

$$logL\left( y \right)=\sum_{i=1}^{n} log\left[ dnorm(\sqrt{y_{i}};\mu_{i} ;\sigma)\frac{1}{2\sqrt{y_{i}+0.5}} \right]$$

where *dnorm* is the density of normal distribution, y_i_= CopS_i_, $\mu_{i}$is the log(CopS_i_+ε_i_ ) predicted by the regression model, ε_i_=1,0.5,0.01, $\sqrt{y_{i}}$=square root transformed CopS_i_, σ= generate using maximum likelihood estimate from fitting the model to the log transformed response or to the root square transformed response.

**R code** to calculate a function obtain normal **log-likelihood for untrasformed response**:

*norm.test<-function(model,y)⎨*

*s<-sqrt(sum(residuals(model)^2/length(residuals(model)))*

*LL<-sum(log(dnorm(y,mean=predict(model),sd=s)))⎬*

*norm.test<-(model,y)*

**R code** to calculate a function obtain lognormal **log-likelihood for trasformed response**:

*norm.log<-function(model,y)⎨*

*t.y<-log(y+ε)*

*s<-sqrt(sum(residuals(model)^2/length(residuals(model)))*

*LL<-sum(log(dnorm(t.y,mean=predict(model),sd=s)*1/y))⎬*

*norm.log<-(model,y)*

**R code** to calculate a function obtain a square root **log-likelihood for trasformed response**:

*norm.sqrt<-function(model,y)⎨*

*t.y<-sqrt(y)*

*s<-sqrt(sum(residuals(model)^2/length(residuals(model)))*

*LL<-sum(log(dnorm(t.y,mean=predict(model),sd=s)*1/(2sqrt(y+0.5))))⎬*

*norm.sqrt<-(model,y)*

Data set is available in S1 Dataset

**Reference**

Weiss,J. (2010) *Statistical Methods in Ecology.* University of North Caroline. <http://www.unc.edu/courses/2010fall/ecol/563/001/docs/lectures/lecture15.htm> [accessed 24 May 2016].
