# Supplementary material for: Generalized structural equations improve sexual-selection analyses
Source: PLoS One. 2017 Aug 15;12(8):e0181305. doi: 10.1371/journal.pone.0181305 (PMC5557364; doi:10.1371/journal.pone.0181305)
Supplement: S6 Text — (DOCX) [file pone.0181305.s006.docx]

**S6 Text**

List of data set to implement SEM and GSEM with STATA and Mplus.

For STATA and Mplus will import the data-set: datiSEM_def.xlsx (in S1 Dataset).
