# Supplementary material for: Generalized structural equations improve sexual-selection analyses
Source: PLoS One. 2017 Aug 15;12(8):e0181305. doi: 10.1371/journal.pone.0181305 (PMC5557364; doi:10.1371/journal.pone.0181305)
Supplement: S1 Table — (DOCX) [file pone.0181305.s007.docx]

**S1 Table.** Complete list of variables and their definitions used in the models GLMs, SEMs and GSEM.

| ***Model's variables name*** | ***Description of variables*** |
| --- | --- |
| *ξ_1_* | Latent variable “Antler shape” is positively related to both number of spellers (*TotS)* and their fluctuating asymmetry (*ASS_T_*). |
| *ASS_T_* | The fluctuating asymmetry of small antler’s spellers. |
| *TotS* | The total number of small and large antler’s spellers. |
| *ξ_1a_* | Latent variable “Dominance rank” is positively correlated to dominance indexes (*Ds, Dom*). |
| *Ds* | The David’s score, *Ds,* (*Gammel et al. 2003*) divided for the total number of bucks of each year. |
| *Dom* | Dominance Index Clutton-Brock et al. (*1979*) divided for the total number of bucks of each year. |
| *η_1_* | Latent variable “Lek attendance” is correlated to lek attendance index (*LA_1_, LA_2_*). |
| *LA_1_* | The number of days in which the animal was present in the lek. |
| *LA_2_* | The total number of days of presence/territory in different locations of the same lek. |
| *η_2_* | Latent variable “Mating success” is related to harem size, courtship behaviour and buck’s copulatory success (*HS, CourtS, CopS* ). |
| *HS* | The mean number of females in a male’s territory. |
| *CourtS* | The fraction of courtship events terminated with a copulation (number of copulations /number of courtship events, for every male). |
| *CopS* | The total copulatory success of the *i*-th buck in one rut. |

**References**

Clutton-Brock, T. H., Albon, S. D., Gibson, R. M. & Guinness, F. E. (1979) The logical stag: adaptative aspects of fighting in red deer (*Cervus elaphus L*.). *Animal behaviour*, 27, 211-225.

Gammel, M. P., De Vries, H., Jennings, D.J., Carlin, C. M.,& Hyden, T. J. (2003) David’s score: a more appropriate dominance ranking method than Clutton-Brock et al.’s index. *Animal Behaviour*, 66, 601-605.
