# Supplementary material for: Generalized structural equations improve sexual-selection analyses
Source: PLoS One. 2017 Aug 15;12(8):e0181305. doi: 10.1371/journal.pone.0181305 (PMC5557364; doi:10.1371/journal.pone.0181305)
Supplement: S2 Table — (DOCX) [file pone.0181305.s008.docx]

**S2 Table**. List of the main papers on copulatory success in lek mating (from 1980 to 2016), with a synthetic description of methods used.

| ***Paper*** | ***Statistical Method*** | ***Description*** |
| --- | --- | --- |
| Clutton-Brock (1988) | a) Correlation  b) Non-parametric test | Dependent variables were tested for normality |
| Apollonio et al. (1989) | LM | Square root transformation of response variable (copulatory success). |
| Goslin & Petrie (1990) | Chi-square test |  |
| Thirgood (1990) | a) Descriptive Statistical analysis.  b) Chi-square test | Dependent variables were tested for normality |
| Balmford et al. (1992) | Spearman’s correlation | The distribution of male reproductive success fitted to a Poisson. |
| Deutsch & Nefdt (1992)) | LM | Log transformation of copulatory success log(copulations+1) |
| Byers et al. (1994) | Pearson’s correlation | Normalized copulatory success using mean number of copulation per rut attained per male aged 1-9 years. |
| Deutsch (1994) | a) Descriptive Statistical analysis  b) Pearson’s correlation |  |
| Marks et al. (1994) | a) Simulations  b) LM | Standardized male mating success. |
| Focardi & Tinelli (1996a) | a) Simulations  b) LM  c) Test for Poissonian process. | 1) CV for standardized copulatory success.  2) The distribution of male copulatory success fitted as a Poisson. |
| Mackenzie et al. (1995) | Theoretical model | Random female mating assumed to be Poisson distributed. |
| Focardi & Tinelli (1996b) | Path analysis with manifest variables | Rank procedure for non-normal variable transformation. |
| Hirth (1997) | a) Descriptive statistical  b) Test of density hypothesis |  |
| Kokko et al. (1998) | LM | Log-trasformation of copulatory success. |
| McElligott et al. (1998) | a) Non parametric-test  b) Sperman’s rank correlation | Rank correlation. |
| Fiske et al. (1998) | a) Sperman’s rank correlation  b) Power of test | Rank correlation. |
| Kokko et al. (1999) |  | Memory of the lekking system as a determinant of the male mating success Power test |
| McElligott et al. (1999) | LM |  |
| Isvaran & Jhala (2000) | Descriptive statistical |  |
| McElligott et al. (2001) | a) Kendall rank-order correlation coefficient  b) Partial rank-order correlation coefficient | Pearson’s correlation to investigate collinearity |
| Rintamaki et al. (2001) | a) GLM  b) Logistic regression | 1) Poisson distribution for response variable, and logarithm as the link function.  2) Response variable transformation from copulation number to a binomial variate (0 or 1). |
| Bro-Jørgensen (2003b) | Non-parametric test | 1) Friedman tests to avoid pseudoreplication.  2) Dunnett’s tests for multiple comparisons |
| Loyau et al. (2007) | GLM | Poisson distribution of response variable and logarithm as the link function. |
| Fricova et al. (2008) | GLMM (Generalized Linear Mixed Models) | Count distribution of response variable and locations as random factor. |
| Bro-Jørgensen (2008) | Logistic Regression Model. | Response variable transformation from copulation number to a binomial variate (0 or 1). |
| Bro-Jørgensen et al. (2011) | REML (Linear Mixed Model) | 1) Model with male ID as random factor  2) Covariates (age) included as quadratic expression in order to allow for the non linear relationship with mating success. |
| Ciuti et al. (2011) | LME (Linear Mixed Effects Model) | 1) Model with male ID as random factor  2) Log-trasformation (copulations + 1) |
| Ciuti et al. (2011b) | LME (Linear Mixed Effects Model) | 1) Data log-transformation of dependent variables  2) Dependent variables were successful tested for normality and homoscedasticity after trasformation. |
| Kervinen et al. (2012) | ZIP (Zero Inflated-Model) | Model with Poisson error distribution. |
| Dakin & Montgomerie (2013) | GLMM (Generalized Linear Mixed Models) | 1. Fourth root trasformed standardized copulation number. 2. Use of ZIP and male ID as random factor. |
| Sardell et al (2014) | a) Correlation  b) LM  c) Chi-square |  |

**References**

Apollonio, M., Festa-Bianchet, M. & Mari, F. (1989) Correlates of copulatory success in a fallow deer Lek. Behav. *Ecol. Sociobiology*, 25, 89-97.

Balmford, A. & Turyaho, M. (1992). Predation risk and lek-breeding in Uganda Kob. *Animal Behaviour*, 44, 117-127.

Byers, J. A., Moodie, J. D. & Hall, N. (1994). Pronghorn females choose vigorous mates. Animal Behaviour, 47, 33-43.

Bro-Jørgensen, J. & Durant, S. M. (2003b) Mating strategies of topi bulls: getting in the centre of attention. *Animal Behaviour*, 65, 585-594.

Bro-Jørgensen J. The impact of lekking on the spatial variation in payoff to resource-defending topi bulls, *Damaliscus lunatus*. Animal Behaviour. 2008; 75: 1229-1234.

Bro-Jørgensen J. Queuing in space and time reduces the lek paradox on an antelope lek. *Ecology & Evolution*. 2011a; 25: 1385-1395.

Ciuti, S. & Apollonio, M. (2011) Do antlers honestly the phenotypic quality of fallow buck (Dama dama) in a lekking population? *Ethology*, 117, 133-144.

Ciuti, S., De Cena, F., Bongi, P. & Apollonio, M. (2011). Benefits of a risky life for fallow deer bucks (*Dama dama*) aspiring to patrol a lek territory *Behaviour*, 148, 435-460.

Clutton-Brock, T.H., Green, D., Hiraiwa-Hasegawa, M. & Albon, S.D., (1988). Passing the buck: resource defence, lek breeding and mate choice in Fallow deer. Behav. *Ecol. Sociobiol*., 23, 281-296.

Dakin, R. & Montgomerie , R. (2013). Eye for an eyespot: how iridescent plumage

ocelli influence peacock mating success. Behavioral Ecology, doi:10.1093/beheco/art045.

Deutsch J.C., Nefdt R.J.C. (1992). Olfactory cues influence female choice in two lek-breeding antelope. Nature 356:596-598

Deutsch, J.C. (1994). Uganda Kob mating success does not increase on larger leks. *Behavioural Ecol. Sociobiol*, 34, 451-459.

Fiske, P., Rintamaki, P. T. & Karvonen, E. (1998). Mating success in lekking males: a meta-analysis. *Behavioural Ecology*, 9, 328-338.

Focardi, S. & Tinelli, A. (1996a). May random processes explain mating success in leks? *Behavioural Processes*, 36, 227-237.

Focardi, S., & Tinelli, A. **(**1996b**).** A structural-equations model far the mating behaviour of bucks in a lek of fallow deer. *Ecology & Evolution*, 8, 413-426.

Fričová B., Bartoš, L., Bartošová, J., Panamá, J., Šustr, P., & Jozífková, E. (2008). Comparison of reproductive success in fallow deer males on lek and single temporary stands. *Folia Zool.* 57, 269-273.

Goslin, L. M & Petrie, M. (1990). Lekking in topi: a consequence of satellite behaviour by small males at hotspots. *Animal Behaviour*, 40, 272-287.

Hirth, D. (1997). Lek breeding in a Texas population of fallow deer (*Dama dama*).American *Midland Naturalist*, 138, 276-289.

Isvaran , K. & Jhala , Y. (2000). Variation in Lekking Costs in Blackbuck (*Antilope cervicapra*): Relationship to Lek-Territory Location and Female Mating Patterns. *Behaviour*, 137,547-563.

Kervinen, M., Rauno V. Alatalo, R. V., Christophe Lebigre, C., Siitari, H. & Soulsbury, D. C. (2012). Determinants of yearling male lekking effort and mating success in black grouse (*Tetrao tetrix*). *Behavioral Ecology*, *ars104,* 1-9.

Kokko, H., Lindstrom, J., Alatalo, R. V. & Rintamaki, P. T. (1998). Queuing for territory positions in the lekking black grouse (*Tetrao tetrix*). *Behavioral Ecology*, *9,* 376-383.

Kokko, H., Rintamaki, P. T., Alatalo, R.V., Hoglund, J., Karvonen, E. & Lundberg, A. (1999). Female choice selects for lifetime lekking performance in black grouse males. *Proc. R. Soc. Lond B*. 266, 2109-2115.

Loyau, A., Gomez, D. Moureau, B., Théry, M., Hart, N. S., Saint Jalme, M., Bennett, A. T. D. (2007). Iridescent structurally based coloration of eyespots correlates with mating success in the peacock. *Behavioural Ecology*, 18, 1123-1131.

Mackenzie, A., Reynolds, J. D., Brown, V. J. & Sutherland, W. J. (1995). Variation in Male Mating Success on Leks. *The American Naturalist*, 145, 633-652.

Marks, A., Deutsch, J.C. & Clutton-Brock, T.H. (1994). Stochastic influences, female copying and the intensity of sexual selection on leks. Journal theor. Biol., 170, 159-162.

McElligott, A. G., Mattiangeli, V., Mattiello, S., Verga, M., Reynolds, C. A. & Hayden, T.J. (1998). Fighting tactics of fallow bucks (*Dama dama*, Cervidae): Reducing the risks of serious conflicts. *Ethology*, 104, 789-803.

McElligott, A. G., O’Neill, K.P. & Hayden, T. J. (1999). Cumulative long-term investment in vocalization and mating success of fallow bucks, *Dama dama. Animal Behaviour,* 57, 1159-1167.

McElligott, A. G., Gammell, M. P., Harty, H. C., Paini, D. R.,Murphy, D. T., Walsh, J. T. & Hayden, T. J. (2001). Sexual size dimorphism in fallow deer (Dama dama): do larger, heavier males gain greater mating success? *Behavioral Ecology and Sociobiology*, 49, 266–272.

Rintamaki, P. T., Hoglund, J., Alatalo, R.V. & Lundberg, A. (2001). Genetic and behavioural estimates of reproductive skew in male fallow deer. *Ann. Zool. Fennici*, 38, 99-109.

Sardell, R. J., Kempenaers, B. & Duvall, E. H. (2014). Female mating preferences and offspring survival: testing hypotheses on the genetic basis of mate choice in a wild lekking bird. *Molecular Ecology*, 23, 933–946

Thirgood, S. J. (1990). Alternative Mating Strategies and Reproductive Success in Fallow Deer. *Behaviour*,Vol. 116, 1-10.
