# Supplementary material for: Generalized structural equations improve sexual-selection analyses
Source: PLoS One. 2017 Aug 15;12(8):e0181305. doi: 10.1371/journal.pone.0181305 (PMC5557364; doi:10.1371/journal.pone.0181305)
Supplement: S3 Table — (DOCX) [file pone.0181305.s009.docx]

**S3 Table**. This table show the complete list of variables name of the models and the respective path coefficients.

| ***Model's variables name*** | ***Path Coefficients*** |
| --- | --- |
| *ξ_1_* |  |
| *ASS_T_* | *λ_1_* |
| *TotS* | *λ_2_* |
| *ξ_1α_* |  |
| *Ds* | *λ_1α_* |
| *Dom* | *λ_2α_* |
| *η_1_* |  |
| *LA_1_* | *λ_3_* |
| *LA_2_* | *λ_4_* |
| *η_2_* |  |
| *HS* | *λ_5_* |
| *CourtS* | *λ_6_* |
| *CopS* | *λ_7_* |
| ***Structural or Causal Model*** | ***Path Coefficients*** |
| *Female Choice Hypothesis* |  |
| *ξ_1_ –> η_1_* | *γ_1_* |
| *η_1_ –> η_2_* | *β_2_* |
| *Male Dominance Hypothesis* |  |
| *ξ_1α_ –> η_1_* | *γ_1α_* |
| *η_1_ –> η_2_* | *β_1_* |
