# Supplementary material for: Generalized structural equations improve sexual-selection analyses
Source: PLoS One. 2017 Aug 15;12(8):e0181305. doi: 10.1371/journal.pone.0181305 (PMC5557364; doi:10.1371/journal.pone.0181305)
Supplement: S4 Table — (DOCX) [file pone.0181305.s010.docx]

**S4 Table.** Summary results models (LM/GLM) analysis

| Model |  | Type response | Predictor variables | | | | | | | | |
| --- | --- | --- | --- | --- | --- | --- | --- | --- | --- | --- | --- |
|  |  |  | *ASS_T_* | *TotS* | *Dom* | *Ds* | *LA_1_* | *LA_2_* | *HS* | *CourtS* |  |
| *LM_1_* |  | Gaussian | -0.097 | -0.059 | -0.268 | 0.064 | 0.146 | -0.026 | **1.573** | **1.328** |  |
| *LM_1,r_* |  | Gaussian |  |  |  |  |  |  | **1.584** | **1.480** |  |
| *LM_2_* |  | Gaussian | 0.003 | -0.010 | -0.017 | 0.003 | 0.024 | -0.004 | **0.158** | **0.587** |  |
| *LM_2,r_* |  | Gaussian |  |  |  |  |  |  | **0.164** | **0.606** |  |
| *LM_3_* |  | Gaussian | -0.006 | -0.012 | -0.015 | 0.002 | 0.027 | -0.004 | **0.182** | **0.818** |  |
| *LM_3,r_* |  | Gaussian |  |  |  |  |  |  | **0.188** | **0.839** |  |
| *LM_4_* |  | Gaussian | -0.019 | -0.014 | -0.008 | -0.001 | 0.031 | -0.003 | **0.226** | **1.450** |  |
| *LM_4,r_* |  | Gaussian |  |  |  |  |  |  | **0.231** | **1.473** |  |
| *LM_5_* |  | Gaussian | 0.010 | -0.015 | -0.033 | 0.006 | 0.035 | -0.006 | **0.263** | **0.734** |  |
| *LM_5,r_* |  | Gaussian |  |  |  |  |  |  | **0.261** | **0.778** |  |
| *GLM_1_* |  | Poisson | 0.051 | **-0.066** | 0.398x10^-5^ | **-0.029** | **0.070** | -0.079 | **0.402** | **1.476** |  |
| *GLM_1,r_* |  | Poisson |  | **-0.061** |  | **-0.030** | **0.046** |  | **0.414** | **1.460** |  |
| *GLM_2_* |  | Neg. Binom. | -0.014 | -0.033 | -0.016 | -0.014 | 0.076 | -0.008 | **0.436** | **1.851** |  |
| *GLM_2,r_* |  | Neg. Binom. |  |  |  |  |  |  | **0.448** | **1.897** |  |
| *GLM_3_* |  | ZIP | 0.051 | **-0.066** | 0.150x10^-4^ | **-0.029** | **0.070** | -0.079 | **0.402** | **1.476** |  |
| *GLM_3,r_* |  | ZIP |  | **-0.061** |  | **-0.030** | **0.046** |  | **0.414** | **1.460** |  |
| *GLM_4_* |  | ZINB | -0.014 | -0.033 | -0.016 | -0.014 | 0.076 | -0.008 | **0.436** | **1.851** |  |
| *GLM_4,r_* |  | ZINB |  |  |  |  |  |  | **0.448** | **1.897** |  |
| *GLM_5_* |  | Hurdle | **0.065** | **-0.054** | -0.046 | -0.004 | 0.043 | 0.000 | **0.335** | 0.282 |  |
| *GLM_5,r_* |  | Hurdle | **0.068** | **-0.025** |  |  |  |  | **0321** |  |  |

Reported values are the unstandardized estimated coefficients. Significant coefficients (P<0.05) are shown in bold. Variable names are: *ASS_T_ =* the fluctuating asymmetry of small antler’s spellers*; TotS =* total number of small and large antler’s spellers*; Dom =* Dominance Index ( Clutton-Brock et al., 1979) divided by the total number of bucks of each year; *Ds =* the David’s score (Gammel et al 2003) divided for the total number of bucks of each year; *LA_1_*=number of days in which the animal was present in the lek. *LA_2_*= total number of days of presence/territory in different locations of the same lek. *HS =* average number of females in a male’s territory; *CourtS* = the fraction of courtship events terminated with a copulation (number of copulations / number of courtship events, for every male). Response variable is *CopS* *=*  total copulatory success of the i-th buck in one rut. The number of observations is the same for all models (N=118).

**References**

Clutton-Brock, T. H., Albon, S. D., Gibson, R. M. & Guinness, F. E. (1979) The logical stag: adaptative aspects of fighting in red deer (*Cervus elaphus L*.). *Animal behaviour*, 27, 211-225.

Gammel, M. P., De Vries, H., Jennings, D.J., Carlin, C. M., Hyden, T. J. (2003) David’s score: a more appropriate dominance ranking method than Clutton-Brock et al.’s index. *Animal Behaviour*, 66, 601-605
