# Supplementary material for: Generalized structural equations improve sexual-selection analyses
Source: PLoS One. 2017 Aug 15;12(8):e0181305. doi: 10.1371/journal.pone.0181305 (PMC5557364; doi:10.1371/journal.pone.0181305)
Supplement: S5 Table — (DOCX) [file pone.0181305.s011.docx]

**S5 Table**. Measured covariances (upper triangle), variances (diagonal show in bold type face) and correlations matrix (lower triangle)in a data set consisting of 118 observations. a) FCH model; b) MDH model.

| *Variables* | *ASS_T_* | *TotS* | *LA_1_* | *LA_2_* | *HS* | *CourtS* | *CopS* |
| --- | --- | --- | --- | --- | --- | --- | --- |
| *ASS_T_* | **4.094** | 0.099 | 0.191 | 0.214 | 0.100 | 0.103 | 0.061 |
| *TotS* | 1.248 | **39.072** | 0.254 | 0.251 | -0.106 | 0.027 | -0.086 |
| *LA_1_* | 2.859 | 11.777 | **54.819** | 0.907 | 0.118 | 0.264 | 0.234 |
| *LA_2_* | 9.103 | 32.985 | 141.255 | **442.782** | 0.067 | 0.295 | 0.185 |
| *HS* | 0.314 | -1.032 | 1.356 | 2.182 | **2.410** | 0.530 | 0.627 |
| *CourtS* | 0.208 | 0.170 | 1.950 | 6.189 | 0.820 | **0.992** | 0.537 |
| *CopS* | 0.640 | -2.774 | 8.952 | 20.090 | 5.031 | 2.766 | **26.717** |

a)

| *Variables* | *Ds* | *Dom* | *LA_1_* | *LA_2_* | *HS* | *CourtS* | *CopS* |
| --- | --- | --- | --- | --- | --- | --- | --- |
| *Ds* | **70.847** | 0.588 | 0.154 | 0.119 | 0.256 | 0.256 | 0.225 |
| *Dom* | 13.008 | **6.909** | -0.043 | -0.048 | 0.215 | 0.104 | 0.053 |
| *LA_1_* | 9.585 | -0.831 | **54.819** | 0.907 | 0.118 | 0.264 | 0.234 |
| *LA_2_* | 21.129 | -2.656 | 141.255 | **442.782** | 0.067 | 0.295 | 0.185 |
| *HS* | 3.346 | 0.879 | 1.356 | 2.182 | **2.410** | 0.530 | 0.627 |
| *CourtS* | 2.148 | 0.271 | 1.950 | 6.189 | 0.820 | **0.992** | 0.537 |
| *CopS* | 9.775 | 0.713 | 8.952 | 20.090 | 5.031 | 2.766 | **26.717** |

b)
