# Supplementary material for: Generalized structural equations improve sexual-selection analyses
Source: PLoS One. 2017 Aug 15;12(8):e0181305. doi: 10.1371/journal.pone.0181305 (PMC5557364; doi:10.1371/journal.pone.0181305)
Supplement: S6 Table — (DOCX) [file pone.0181305.s012.docx]

**S6 Table.** Mean, variance, and kurtosis, for residuals distributions of linear models with transformed response variable (*x=CopS*).

| *Model* | *Transformation* | *Mean* | *Var* | *Kurtosis* |
| --- | --- | --- | --- | --- |
| *LM_2_* | *log(x+1)* | 0.56 | 15.15 | 42.18 |
| *LM_2,r_* | *log(x+1)* | 0.59 | 16.12 | 42.06 |
| *LM_3_* | *log(x+0.5)* | 0.60 | 15.59 | 40.49 |
| *LM_3,r_* | *log(x+0.5)* | 0.63 | 16.54 | 40.82 |
| *LM_4_* | *log(x+0.1)* | 0.60 | 16.95 | 33.53 |
| *LM_4,r_* | *log(x+0.1)* | 0.62 | 17.79 | 36.69 |
| *LM_5_* | *x ^0.5^* | 0.34 | 15.53 | 41.05 |
| *LM_5,r_* | *x ^0.5^* | 0.37 | 14.79 | 40.74 |
