# Supplementary figures and images for: Generalized structural equations improve sexual-selection analyses
Source: PLoS One. 2017 Aug 15;12(8):e0181305. doi: 10.1371/journal.pone.0181305 (PMC5557364; doi:10.1371/journal.pone.0181305)

Preserve of Castelporziano

Rome

Lek

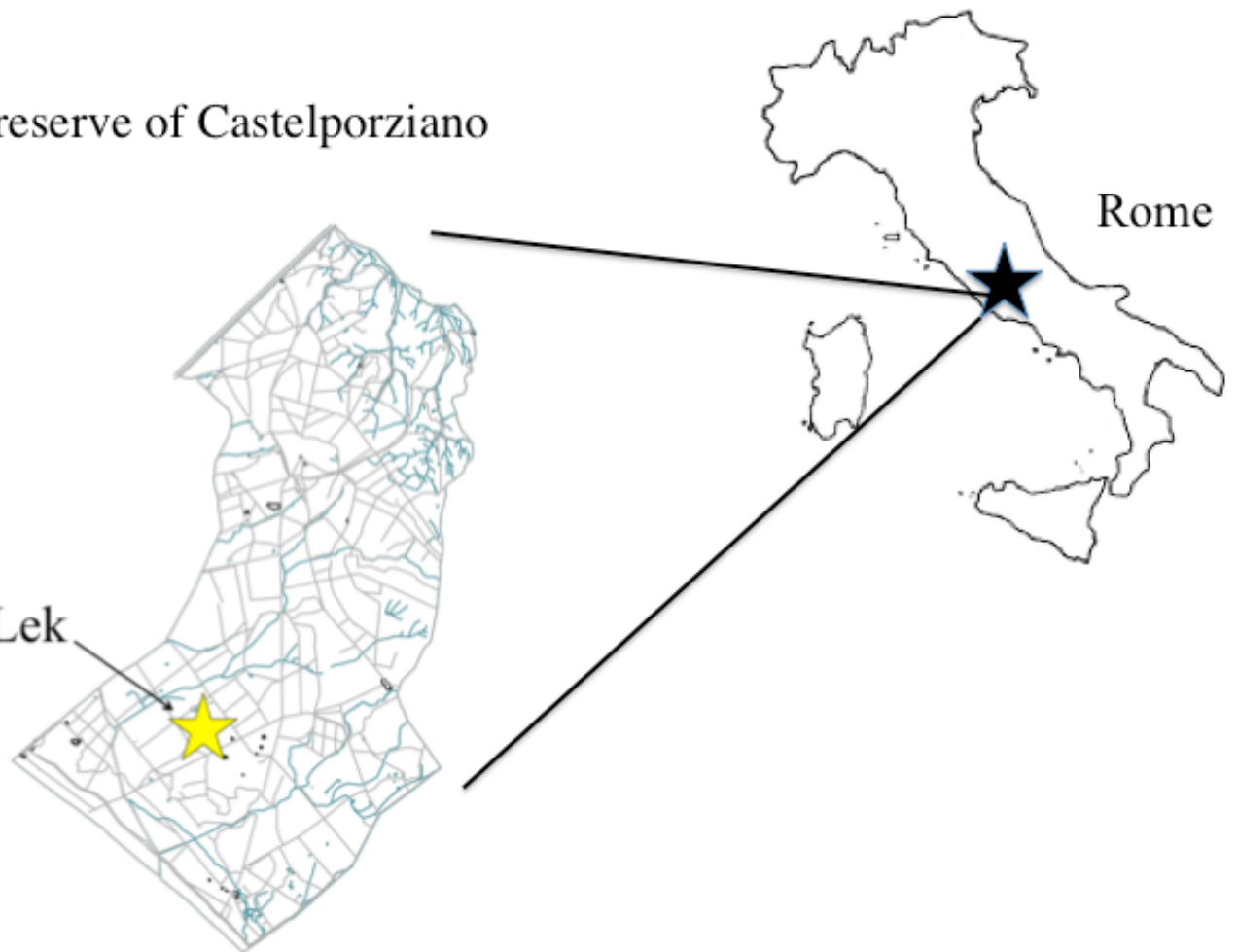

Supplement: S1 Fig — (PDF) [file pone.0181305.s013.pdf]
