# Supplementary material for: Generalized structural equations improve sexual-selection analyses
Source: PLoS One. 2017 Aug 15;12(8):e0181305. doi: 10.1371/journal.pone.0181305 (PMC5557364; doi:10.1371/journal.pone.0181305)
Supplement: S2 Fig — (PDF) [file pone.0181305.s014.pdf]

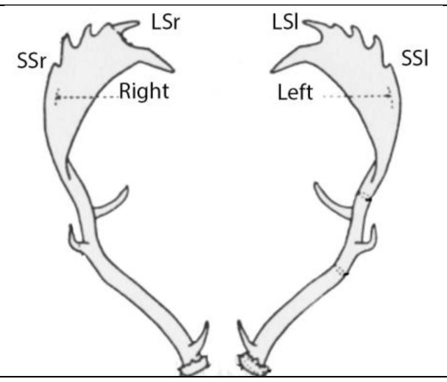

a

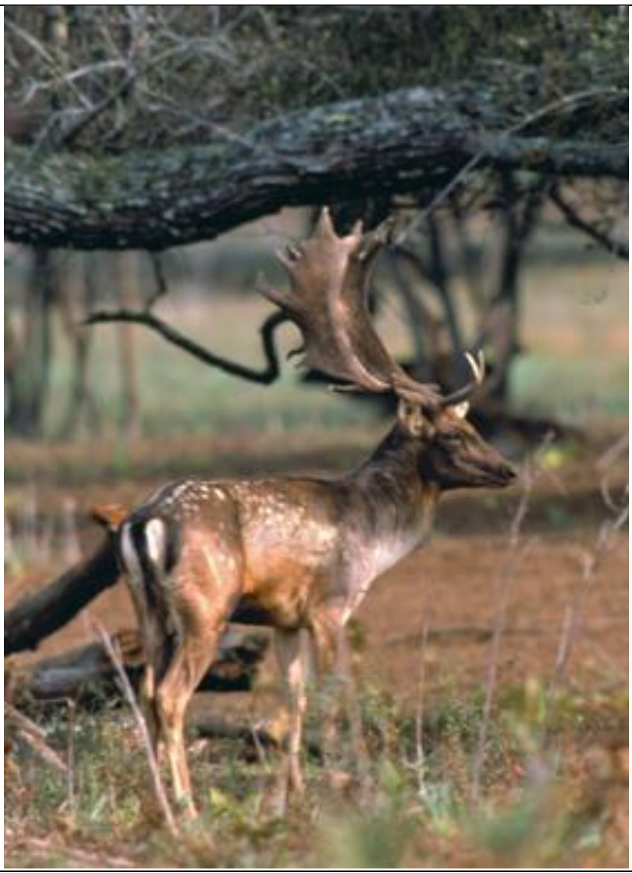

b

S1 Figure 2. a) Let us define SS_r and LS_r the small and large spellers of the right antler, respectively and SS_l and LS_l those of the left antler and consequently RS_T like the right total spellers and $LeftS_T$ the left total spellers. We may compute global measures for antlers size:
$SS_T = SS_r + SS_l$, $LS_T = LS_r + LS_l$, $LeftS_T = LS_l + SS_l$, $RS_T = LS_r + SS_r$, $TotS = RS_T + LeftS_T$ for total spellers (small and large).b) Male fallow deer in Castelporziano.

Model's variables name	***Description of variables***
ξ_1	Latent variable "Antler shape" is positively related to both number of spellers (*TotS)* and their fluctuating asymmetry (ASS_T).
ASS_T	The fluctuating asymmetry of small antler's spellers.
TotS	The total number of small and large antler's spellers.
ξ_{1a}	Latent variable "Dominance rank" is positively correlated to dominance indexes (*Ds, Dom*).
Ds	The David's score, *Ds,* (*Gammel et al 2003*) divided for the total number of bucks of each year.
Dom	Dominance Index Clutton-Brock et al. (1979) divided for the total number of bucks of each year.
η_1	Latent variable "Lek attendance" is correlated to lek attendance index (LA_1, LA_2).
LA_1	The number of days in which the animal was present in the lek.
LA_2	The total number of days of presence/territory in different locations of the same lek.
η_2	Latent variable "Mating success" is related to harem size, courtship behaviour and buck's copulatory success (*HS, CourtS, CopS*).
HS	The mean number of females in a male's territory.
CourtS	The fraction of courtship events terminated with a copulation (number of copulations /number of courtship events, for every male).
CopS	The total copulatory success of the *i*-th buck in one rut.

References

Clutton-Brock, T. H., Albon, S. D., Gibson, R. M. & Guinness, F. E. (1979) The logical stag: adaptative aspects of fighting in red deer (*Cervus elaphus L.*). *Animal behaviour*, 27, 211-225.

Gammel, M. P., De Vries, H., Jennings, D.J., Carlin, C. M.,& Hyden, T. J. (2003) David’s score: a more appropriate dominance ranking method than Clutton-Brock et al.’s index. *Animal Behaviour*, 66, 601-605.
