# Supplementary material for: Generalized structural equations improve sexual-selection analyses
Source: PLoS One. 2017 Aug 15;12(8):e0181305. doi: 10.1371/journal.pone.0181305 (PMC5557364; doi:10.1371/journal.pone.0181305)
Supplement: S3 Fig — (PDF) [file pone.0181305.s015.pdf]

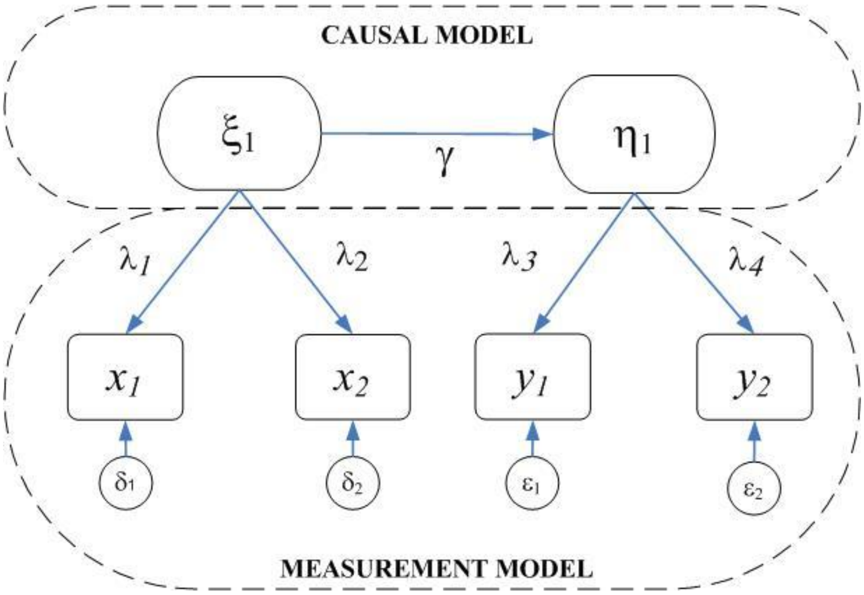


**S3 Figure.** The SEM model of *2S3 eqn*. The causal model is formed by two latent variables ($\xi_1$ exogenous and $\eta_1$, endogenous), while $\gamma$ represents the causal link according to the arrow's direction. Measurement model: $x_1$, $x_2$ (exogenous) and $y_1$, $y_2$ (endogenous) are manifest variables, $\delta_1$, $\delta_2$ ,$\varepsilon_1$ and $\varepsilon_2$ are their error terms, $\lambda_1$, $\lambda_2$, $\lambda_3$ and $\lambda_4$ are the factors loading.
